# Application Value and Research Frontiers of Immunotherapy in Glioblastoma: A Bibliometric and Visualized Analysis

**DOI:** 10.32604/or.2025.069442

**Published:** 2025-12-30

**Authors:** Kun Deng, Jianliang Huang, Danyang Li, Wei Gao, Minghua Wu, Mingsheng Lei

**Affiliations:** 1 Institute of Modern Biomedical Research, Zhangjiajie College, Zhangjiajie, 427000, China; 2 The Key Laboratory of Carcinogenesis of the Chinese Ministry of Health, Cancer Research Institute, Xiangya School of Medicine, Central South University, Changsha, 410078, China; 3 The Key Laboratory of Carcinogenesis and Cancer Invasion of the Chinese Ministry of Education, Cancer Research Institute, Xiangya School of Medicine, Central South University, Changsha, 410078, China; 4 Xiangya School of Public Health, Xiangya School of Medicine, Central South University, Changsha, 410078, China; 5 Department of Pulmonary and Critical Care Medicine, Zhangjiajie Hospital Affiliated to Hunan Normal University, Zhangjiajie, 427000, China

**Keywords:** Glioblastoma, immunotherapy, chimeric antigen receptor-T, bibliometric

## Abstract

**Background:**

Glioblastoma (GBM) prognosis has seen little improvement over the past two decades. While immunotherapy has revolutionized cancer treatment, its impact on GBM remains limited. To characterize the evolving research landscape and identify future directions in GBM immunotherapy, we conducted a comprehensive bibliometric review.

**Methods:**

All literature related to immunotherapy in GBM from 1999 to 2024 was collected from the Web of Science Core Collection. CtieSpace and VOSviewer were used to conduct bibliometric analysis and visualize the data.

**Results:**

Bibliometric analysis identified 5038 publications authored by 23,335 researchers from 4699 institutions across 96 countries/regions, published in 945 journals. The United States produced the highest number of publications, while Switzerland achieved the highest average citation rate. Duke University led in institutional output and citations. John H Sampson was the most productive author, and Roger Stupp was the most cited. *Frontiers in Immunology* published the most papers, while *Clinical Cancer Research* was the most cited journal. Research focus centered on adoptive T cell therapy, particularly chimeric antigen receptor (CAR)-T cells with 572 dedicated publications. Within CAR-T research for GBM, the University of Pennsylvania was the leading institution, *Frontiers in Immunology* the predominant journal, and Christine E Brown (City of Hope National Medical Center) was the most prolific and cited author.

**Conclusions:**

There has been a growing interest in GBM immunotherapy over past decades. The United States is the dominant contributor. CAR-T therapy represents the primary research focus. Emerging strategies like chimeric antigen receptor-modified natural killer (CAR-NK) cells, chimeric antigen receptor-engineered macrophages (CAR-M), and cytomegalovirus-specific T cell receptor (CMV-TCR) T cells are gaining prominence, aiming to address limitations in antigen recognition inherent to CAR-T therapy for GBM.

## 1 Introduction

Glioblastoma (GBM) is the most prevalent and fatal form of brain cancer, accounting for approximately 48% of intracranial tumors [1,2]. The standard therapeutic approach aims to extend overall survival, primarily through surgical, followed by adjuvant chemotherapy and radiotherapy [3,4]. Even with this comprehensive treatment, the median survival of GBM patients remains approximately 14 months, with less than 30% surviving beyond two years after diagnosis [5]. GBM is characterized by an aggressive phenotype and frequently infiltrates the surrounding normal brain tissue, making complete surgical excision challenging [6–8]. Furthermore, the hypoxic tumor microenvironment (TME) and blood-brain barrier (BBB) pose significant challenges for radiotherapy and chemotherapy [9,10]. Upon recurrence, the expected survival time of GBM patients drops drastically to 5–10 months [11]. Therefore, the development of innovative therapeutic strategies for GBM is urgently needed.

GBM treatment is complicated by its distinct biological features and the complex TME [12,13]. Although numerous clinical trials have evaluated the efficacy of innovative targeted therapeutic agents against GBM, most have failed to demonstrate significant clinical benefits. These limitations are primarily attributed to the high heterogeneity of GBM, combined with a lack of therapeutic agents that possess both high biological potency and sufficient BBB penetration [14,15]. Tumor-treating fields (TTFields), an innovative therapeutic modality, have demonstrated the ability to prolong survival among patients with newly diagnosed GBM. However, its application is limited by high cost and stringent adherence requirements [16]. Recently, immunotherapy has achieved notable advances in the treatment of GBM. Compared with other treatment methods, immunotherapy harnesses the body’s immune defenses to combat tumors, primarily through immune checkpoint blockade, therapeutic vaccines, adoptive cell therapy, monoclonal antibodies, or oncolytic viruses [17–19]. These strategies offer the potential to remodel the immunosuppressive TME of GBM and improve therapeutic outcomes.

Before 2015, the central nervous system (CNS) was regarded as an immune-privileged system [20]. In 2015, Louveau et al. [21] discovered functional meningolymphatic vessels, which greatly advanced our understanding of immunological activity within the CNS. Immune cells act as sentinels within the CNS and play vital roles in the maintenance and restoration of brain function. Immune cells are localized in specialized immunological niches (such as meninges, choroid plexus, perivascular spaces), where they are continuously exposed to CNS-derived signals conveyed by the intracranial interstitium, cerebrospinal fluid and lymphatic drainage. This exposure facilitates their migration into the CNS to execute immunological functions [22]. Insights into these mechanisms have established a foundation for novel immunotherapeutic strategies against GBM and offer promising avenues for clinical intervention [23]. Although many studies are examining GBM immunotherapy, most applications remain in the pre-clinical stage. Moreover, the explosive growth in the number of publications may prevent researchers from understanding the crucial advancements and discerning future trajectories in the field of GBM immunotherapy. Therefore, it is essential to identify research hotspots and emerging trends in GBM immunotherapy field through bibliometric analysis.

Bibliometrics is an innovative methodology for synthesizing knowledge, focusing on the statistical and qualitative characteristics of scholarly publications and uncovering important trends in research domains [24,25]. As the scientific literature expands, bibliometric analysis has become increasingly vital in evaluating research landscapes [26]. To the best of our knowledge, there are few systematic and comprehensive bibliometric analyses of GBM immunotherapy. Previous studies have not performed further bibliometric analyses of the research hotspots and frontiers in the field. This study aimed to perform a bibliometric analysis focusing on the output volume, major contributing countries, academic institutions, journals, and leading researchers in GBM immunotherapy since the beginning of the 21st century. We sought to summarize the current research priorities and identify the underlying challenges. These findings are expected to guide future research directions in GBM immunotherapy.

## 2 Materials and Methods

### 2.1 Data Collection

**Figure 1:**
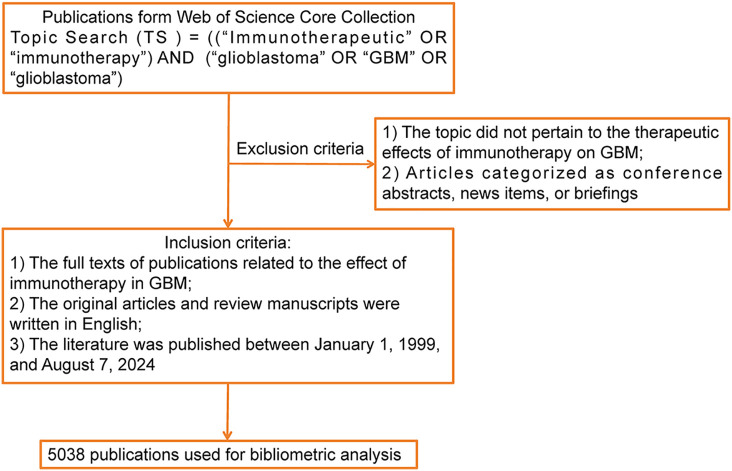
Flowchart of data collection in this study

We performed a search for scholarly publications related to immunotherapy in GBM using the Web of Science Core Collection (WoSCC, Central South University Purchase Edition) database from 01 January 1999 to 07 August 2024. The search terms were precisely defined to encompass the following: Topic Search (TS) = (“Immunotherapeutic” OR “immunotherapy”) AND TS = (“glioblastoma” OR “GBM” OR “glioblastoma”). The literature selection process adhered to three inclusion criteria: (1) the full texts of publications focusing on the effect of immunotherapy in GBM were available; (2) the original articles and reviews were English manuscripts; and (3) the date of publication was between 01 January 1999 and 07 August 2024. Publications were excluded by applying the following criteria: (1) the topic did not pertain to the therapeutic effects of immunotherapy in GBM and (2) articles categorized as conference abstracts, news items, or briefings were excluded from the study, which are not considered high-caliber evidence sources because of their lack of rigorous peer-review and comprehensive details on research methodologies and outcomes. A rigorous manual screening process was employed to filter duplicate and irrelevant studies. Two independent reviewers screened the titles and abstracts of selected articles. Adhering to these stringent criteria, we were able to pinpoint and dissect pivotal research and prevailing trends within the domain of interest with great precision. We exported a dataset of 5038 records in plain-text format encompassing complete records and their cited references to facilitate subsequent analyses and visualization. A detailed flowchart of data collection is presented in Fig. 1.

In addition, we performed a refined literature search focusing on research hotspots in GBM immunotherapy. The search strategy was defined as: TS = (“glioblastoma” OR “GBM”) AND TS = (“chimeric antigen receptor” OR “CAR-T” OR “engineered T-cell receptor” OR “TCR-T”). The same inclusion and exclusion criteria, screening processes, and data extraction procedures were applied as described above.

### 2.2 Data Analysis

GraphPad Prism (version 6; GraphPad Software, Boston, MA, USA) was performed to assess and chart the progression of annual publication volumes as well as the publication trends from various countries. VOSviewer (version 1.6.20, https://www.vosviewer.com (accessed on 01 June 2025)) is a Java-based tool developed by Nees Jan van Eck and Ludo Waltman, who are from the Centre for Science and Technology Studies (CWTS) of Leiden University, the Netherlands. CiteSpace (version 6.2. R4, https://citespace.podia.com (accessed on 01 June 2025)) is developed by Chaomei Chen from the College of Computing and Informatics, Drexel University, Philadelphia, PA, USA [27]. In this study, CiteSpace was primarily employed to conduct co-citation analysis, burst analysis, and detect emerging trends/bursts over time, visualizing the evolution of research fields. VOSviewer was mainly used to construct and visualize bibliometric networks based on co-occurrence data, specifically mapping keyword co-occurrence and author collaboration networks to reveal the structure and scope of the research landscape. These tools provided complementary perspectives; CiteSpace excels at identifying temporal patterns and research frontiers, while VOSviewer effectively handles large-scale network visualizations for understanding domain structure.

## 3 Result

### 3.1 The Global Trends of Annual Publications and Citations

**Figure 2:**
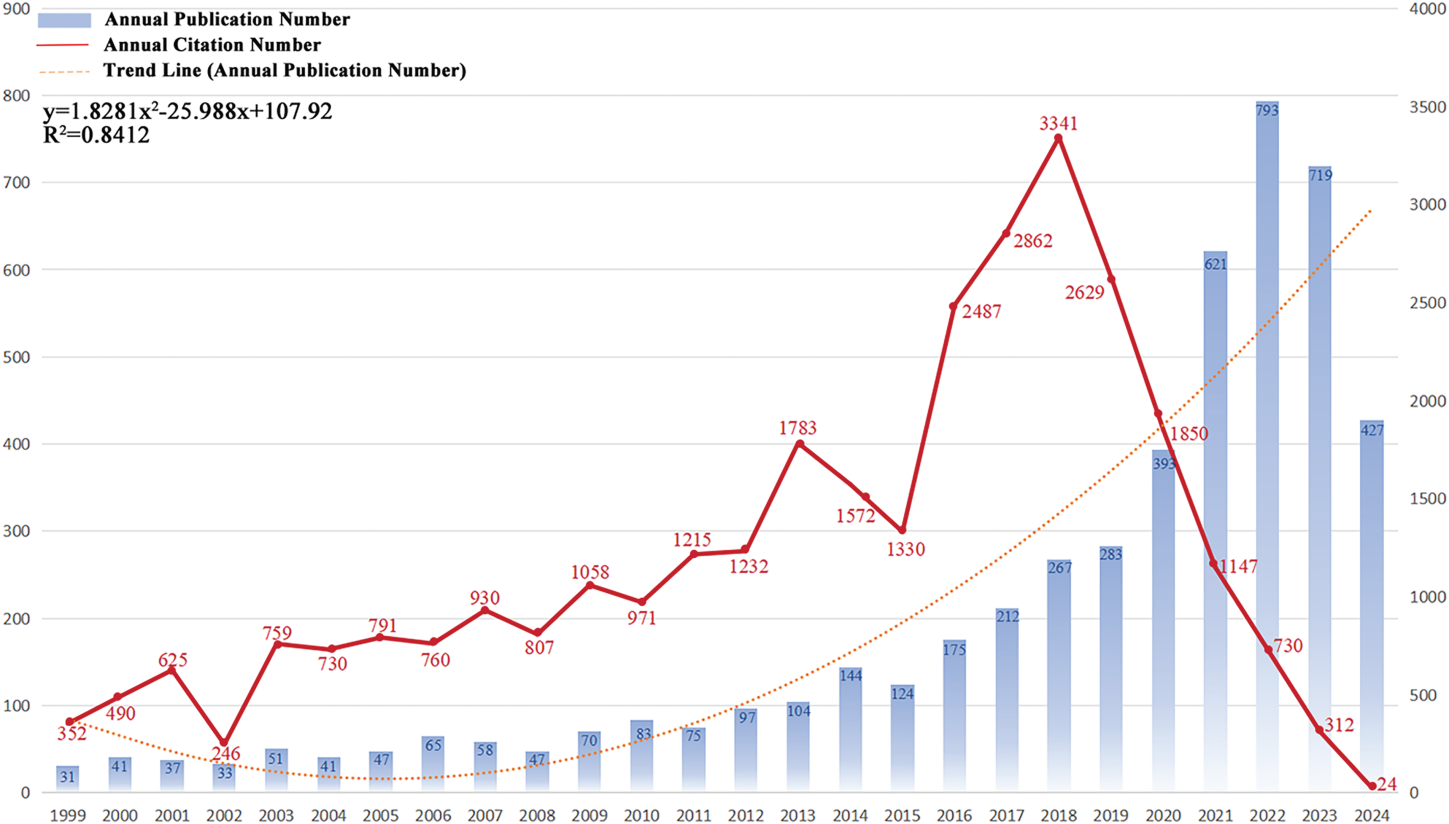
Trends in the growth of publications and the number of citations. The number of publications and citation frequency for each year from 1999 to January 2024 showed a steady growth trend. y = 1.8419x^2^ − 25.988x + 106.54, R^2^ = 0.8415; x is the number of years since 1999, y is the predicted number of publications, and R^2^ indicates the model’s goodness-of-fit

We performed a systematic literature search for immunotherapy applications to GBM using the WoSCC database. A total of 5038 publications were identified, comprising 3637 original articles and 1401 reviews. These publications were published in 945 journals and authored by 23,335 researchers from 4699 institutions across 96 countries and regions. Fig. 2 shows the annual number of publications, publication trends, and citation frequencies in the field of GBM immunotherapy between 1999 and 2024. In summary, the annual volume of publications related to GBM immunotherapy steadily increased between 1999 and 2022, indicating a strong and enduring research trajectory. Annual publications were fitted to a second-order polynomial model (y = 1.8419x^2^ − 25.988x + 106.54, R^2^ = 0.8415, where x is the number of years since 1999, y is the predicted number of publications and R^2^ indicates the model’s goodness-of-fit). However, a slight decrease in annual publications was observed in 2023 compared to 2022, which may be attributed to various factors, such as changes in research funding, shifts in research priorities, or natural fluctuations in publication cycles. Additionally, between 1999 and 2018, the annual citation frequency displayed minor recurring peaks at approximately 1–2 years intervals. These periodic fluctuations may reflect factors such as seminal breakthroughs, evolving research methodologies, shifts in topic focus, and heightened activity in the academic community. Annual citations peaked in 2018, with 3341 citations. Since then, citation counts have gradually declined, which is potentially influenced by citation half-life. In summary, these bibliometric trends highlight the dynamic evolution of GBM immunotherapy research, which suggests that future research directions may be influenced by a confluence of factors, including funding availability, research innovation, and evolving academic discourse. By elucidating these key findings, we aimed to provide a structured perspective on how these trends can shape the future trajectory of research in this field.

### 3.2 Countries and Institutions

**Figure 3:**
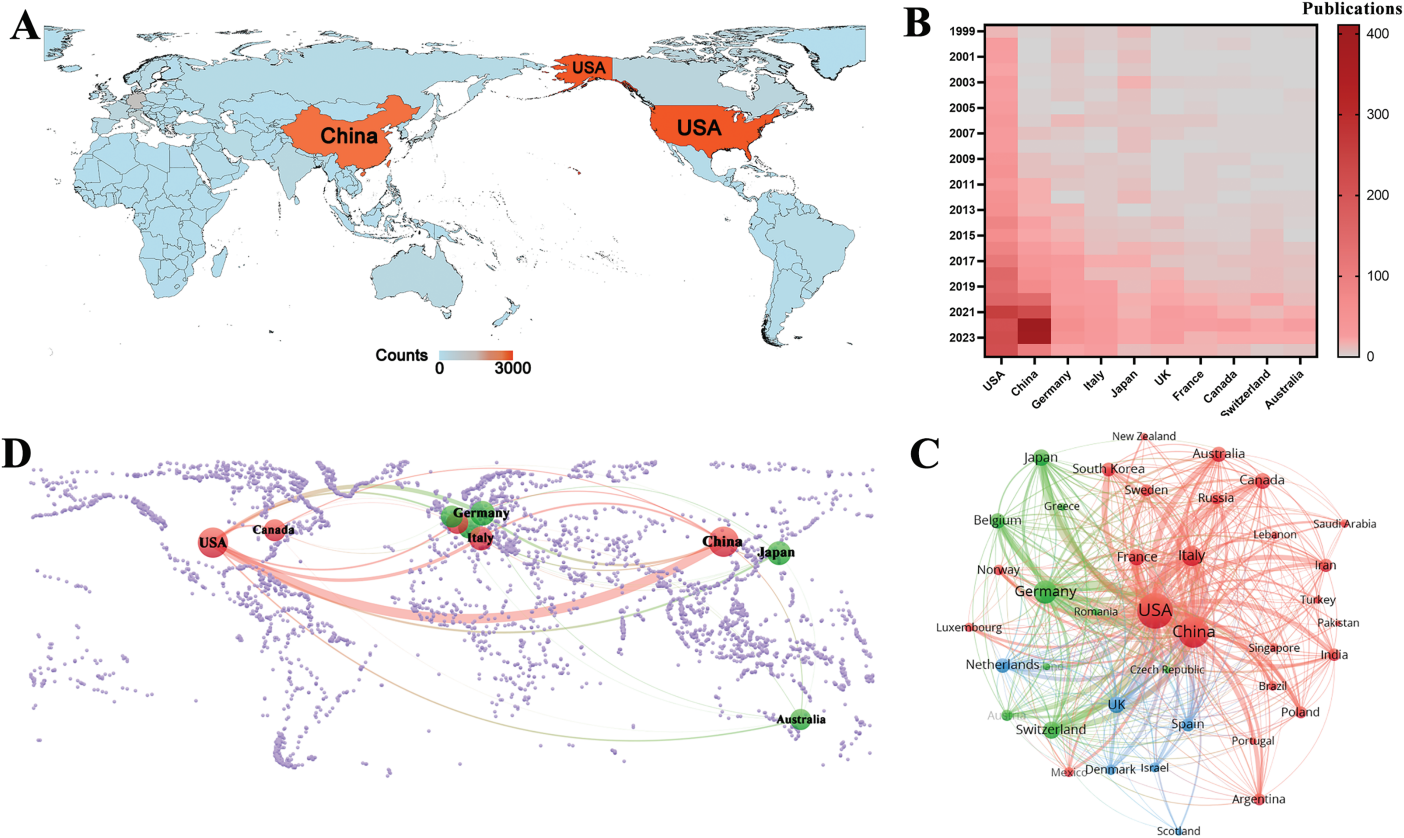
Analysis of country/region. (**A**) Geographical distribution of global output. The volume of publications is represented by color variation; (**B**) Year-by-year output of TOP 10 high-productivity countries; (**C**) Visual cluster analysis of cooperation among countries. The nodes of different colors represent the countries/regions with different clusters, and the thickness of the lines indicates how closely countries cooperate; (**D**) Geographical distribution and the co-authorship network of the top 10 productive countries

This study identified 5038 publications co-authored by investigators from 4699 institutions across 96 countries and regions between 1999 and 2024. Fig. 3A shows the geographical distribution of these countries, with the publication counts represented by varying colors. The visualization clearly indicates that the United States of America (USA) and China were the most active countries in the field. For a detailed overview, Table 1 ranks the top ten countries and regions by publication volume. As shown in Table 1, Switzerland may not have published at a high volume, but the country led in citations per publication (13.96), suggesting that the papers from Swiss institutions were of superior quality. In contrast, China ranked second in the number of publications, but the number of citations per publication (1.63) was low, suggesting that the quality of published papers was generally low. Fig. 3B shows the annual output of the top ten high-productivity countries. Overall, the number of publications across countries has increased annually. Notably, prior to 2020, the USA consistently published the highest number of articles annually in the field of GBM immunotherapy. However, between 2021 and 2023, China demonstrated explosive growth in annual publications, surpassing that of the USA. This was attributed to the fact that the Chinese government introduced a series of robust policies to support the development of immunotherapy (notably cell therapy) between 2017 and 2018. These policies, in turn, significantly stimulated the enthusiasm of Chinese researchers for conducting research in this field. This observation also raised the possibility that a rapid increase in the number of publications may have occurred concurrently with a decline in the average quality of these publications. Fig. 3C illustrates the network of international collaborative ties in research on immunotherapy and GBM. The nodes correspond to countries and regions and the line thickness between them is directly proportional to the degree of collaboration. Fig. 3D shows the geographical distribution and collaborative relationships among the top ten countries/regions. China cooperated closely with the USA, which may explain the rapid increase in the number of publications from China.

**Table 1:** The top 10 countries contributing to publications in the immunotherapy of GBM

Rank	Country	Publications	Citations	Citation per publication
1	USA	2059	22,490	10.92
2	China	1682	2738	1.63
3	Germany	406	3537	8.71
4	Italy	246	1096	4.46
5	Japan	227	2556	11.26
6	UK	170	929	5.46
7	France	137	764	5.58
8	Canada	123	1327	10.79
9	Switzerland	121	1689	13.96
10	Australia	102	1089	10.68

**Figure 4:**
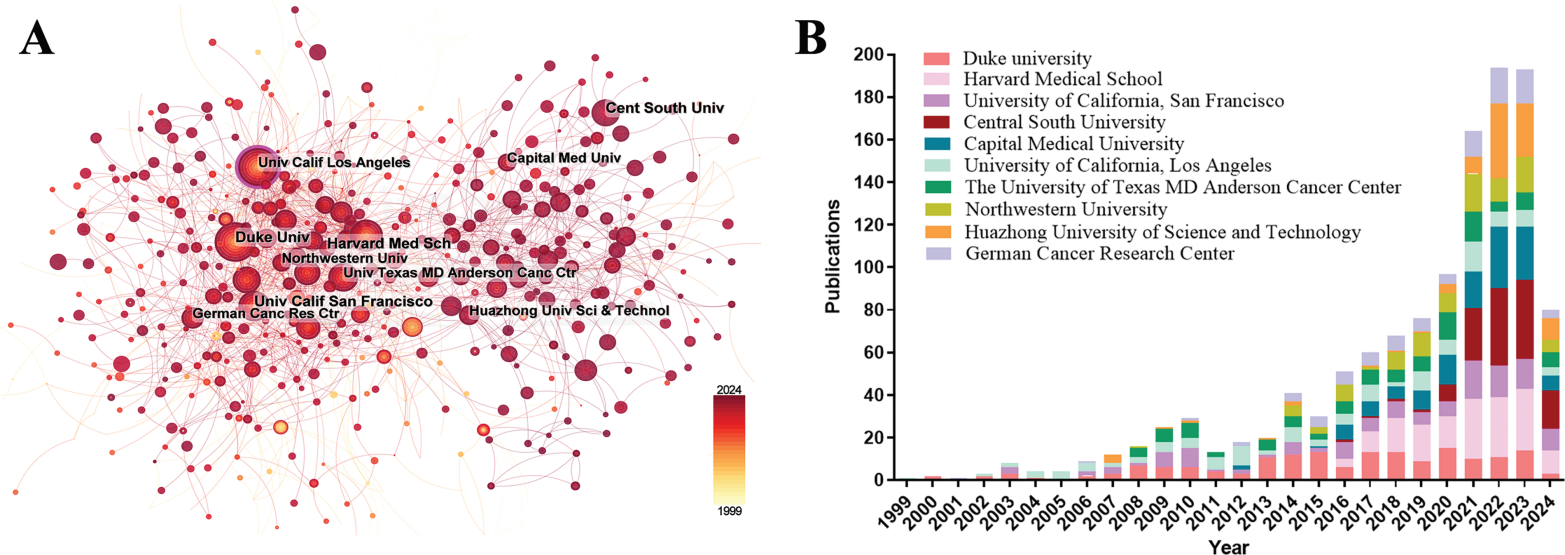
Analysis of institutional contributions and collaborative networks in the field of immunotherapy for GBM. (**A**) Visual cluster analysis of cooperation among institutions. The nodes of different colors represent the institutions with different clusters, and the thickness of the lines indicates how closely institutions cooperate; (**B**) Year-by-year output of TOP 10 high-productivity institutions

Among the top ten institutions ranked by publication output, over half of the institutions (n = 6) were located in the USA, followed by China with three and Germany with one (Table 2). Duke University published the most papers (169 papers, 4327 citations, 25.60 citations/paper). Harvard Medical School (158 papers, 1467 citations, 9.28 citations/paper) ranked second, the University of California, San Francisco (129 papers, 1961 citations, 15.20 citations/paper) ranked third, Central South University (128 papers, 246 citations, 1.92 citations/paper) ranked fourth, and Capital Medical University (124 papers, 192 citations, 1.55 citations/paper) ranked fifth. The proportion of publications by institution was consistent with the proportion of publications by country. Specifically, although Chinese institutions produced a considerable volume of publications, there was a noticeable discrepancy in the citations per publication metric. On average, this metric was significantly lower for Chinese publications than for publications from American institutions. A visualization of the institutional collaboration network is depicted in Fig. 4A. Within this network visualization, the size of each node corresponds to the publication output of the respective institutions. The nodes central to the network are distinguished by pink outer rings, signifying their pivotal intermediary roles. The institution with the highest betweenness centrality (BC) value was the University of California, Los Angeles with a BC value of 0.14. This suggests that the University of California, Los Angeles, has contributed substantially to the advancement and application of immunotherapy in GBM. The temporal distribution of publications, highlighting the output of various institutions across distinct periods, was also examined, offering insights into the evolving research landscape (Fig. 4B). Before 2019, American institutions, particularly Duke University, dominated the field with consistent and high output. However, after 2020, Chinese institutions rose to prominence, notably Central South University and Huazhong University of Science and Technology, which quickly became frontrunners in the annual volume of publications.

**Table 2:** The top 10 institutions contributed to publications in the immunotherapy of GBM

Rank	Institution	Country	Publications	Citations	Citation per publication
1	Duke university	USA	169	4327	25.60
2	Harvard Medical School	USA	158	1467	9.28
3	University of California, San Francisco	USA	129	1961	15.20
4	Central South University	China	128	246	1.92
5	Capital Medical University	China	124	192	1.55
6	University of California, Los Angeles	USA	121	2362	19.52
7	The University of Texas MD Anderson Cancer Center	USA	105	2408	22.93
8	Northwestern University	USA	98	948	9.67
9	Huazhong University of Science and Technology	China	94	102	1.09
10	German Cancer Research Center	German	93	857	9.22

### 3.3 Authors

**Figure 5:**
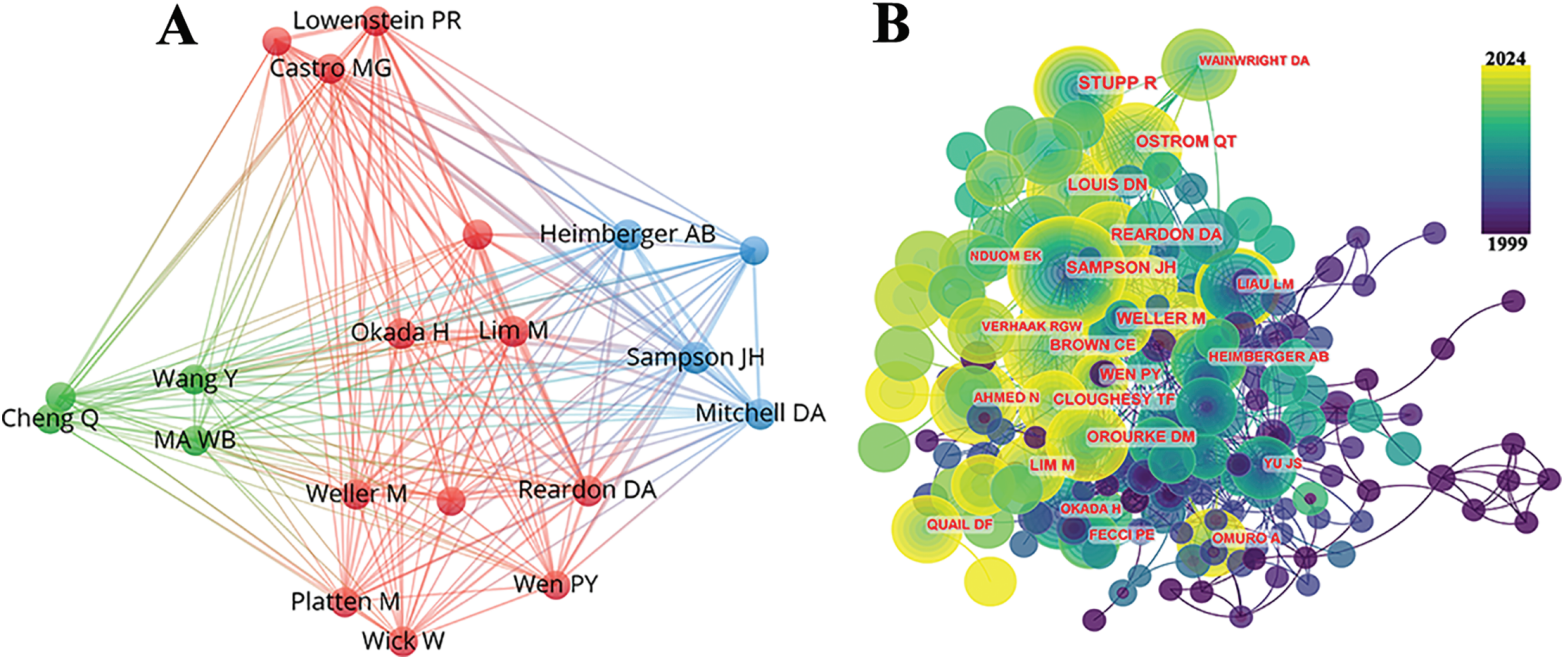
Contributions of the authors of investigations on the immunotherapy of GBM. (**A**) Top 10 author collaboration network chart for publications on immunotherapy of GBM; (**B**) Top 10 most co-cited and cited authors

Table 3 lists the top 10 most prolific authors who have published papers on immunotherapy in GBM. Collectively, these authors published 523 studies, accounting for 10.32% of all publications in the field. The most prolific author was John H Sampson with 82 papers, followed by Lim Michael with 62, and Okada Hideho with 59. Notably, among the leading authors, nine were affiliated with American institutions, whereas one was based in China. The collaborative relationships between the authors are shown in Fig. 5A. Fig. 5B and Table 3 present the top ten authors with the highest co-citation counts. Sixty-two authors were cited more than 200 times, indicating that their research was highly respected and influential within the field. In the visualization network, the largest nodes corresponded to the most frequently cited authors, including Roger Stupp with 1590 citations, David N. Louis with 886, and David A. Reardon with 868.

**Table 3:** The top 10 authors and co-cited authors in the immunotherapy of GBM

Rank	Author	Count	Location	Co-cited author	Citation
1	Sampson John H	82	USA	Stupp Roger	1590
2	Lim Michael	62	USA	Louis David N	886
3	Okada Hideho	59	USA	Reardon David A	868
4	Heimberger Amy B	57	USA	Quinn T Ostrom	867
5	Mitchell Duane A	57	USA	Sampson John H	830
6	Reardon David A	46	USA	Weller Michael	739
7	Castro Maria G	43	USA	Christine E Brown	652
8	Weller Michael	40	USA	Wen Patrick Y	533
9	Lowenstein, Pedro Ricardo	40	USA	Liau Linda M	524
10	Zhu Hua	37	China	Cloughesy Timothy F	499

### 3.4 Journals

**Figure 6:**
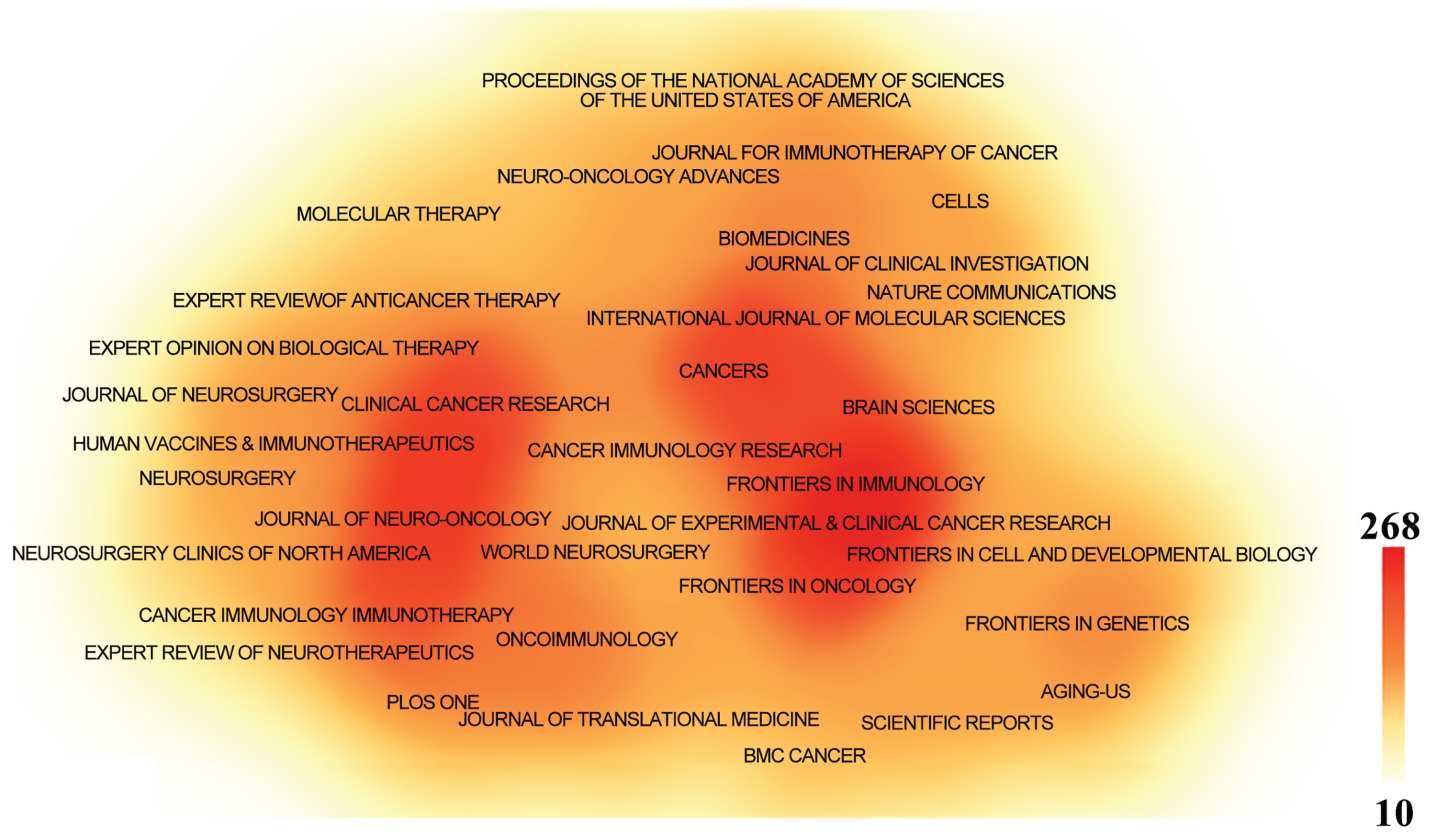
The density map of journals in immunotherapy of GBM. The journals with several publications ≥10

**Figure 7:**
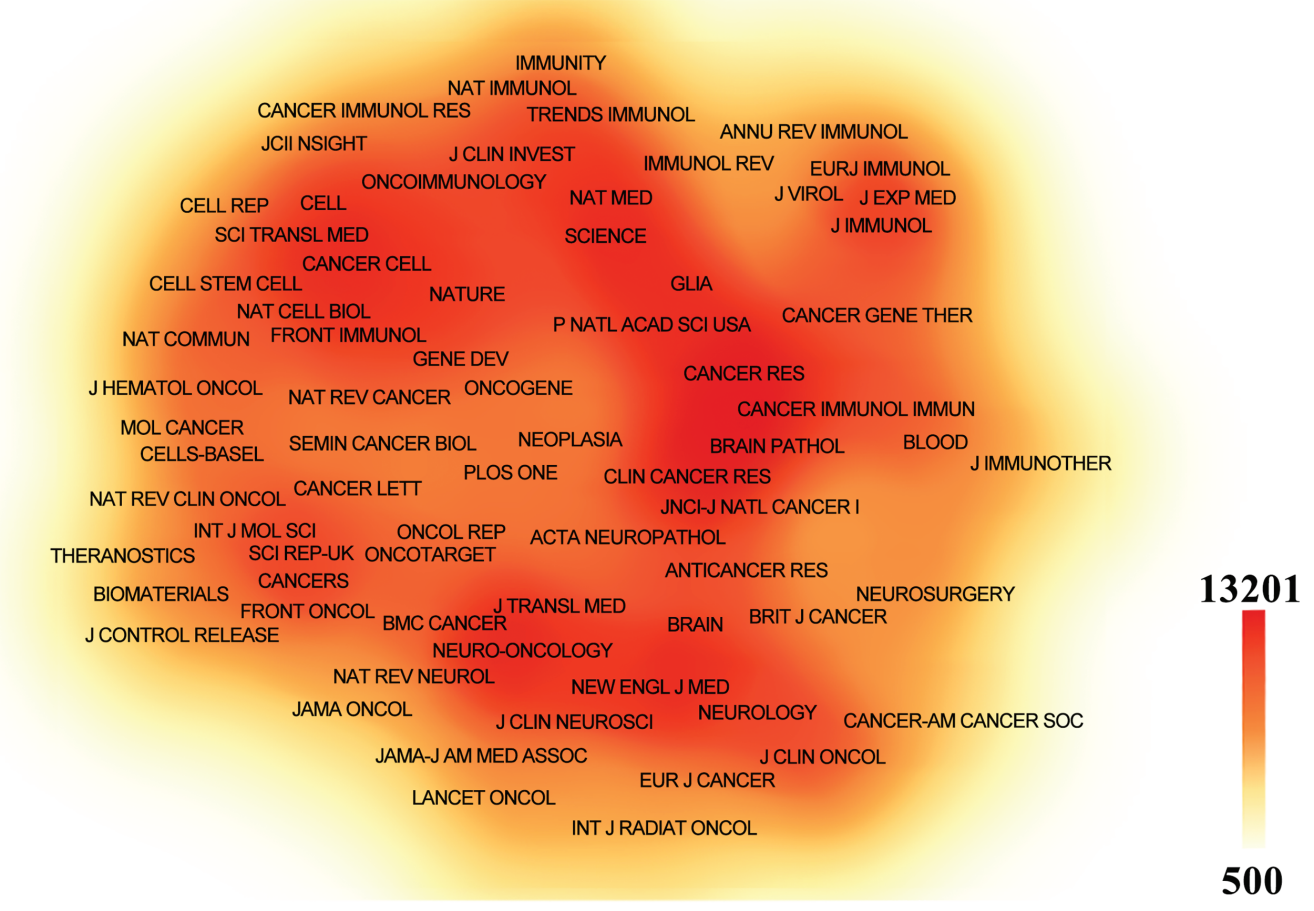
The density map of co-cited journals in immunotherapy of GBM. The co-cited journals with citations ≥500

Papers related to GBM immunotherapy has been published in 945 journals. As shown in Table 4, *Frontiers in Immunology* published 268 papers, followed by *Frontiers in Oncology* with 167, *Cancers* with 157, the *Journal of Clinical Cancer Research* with 114, and *Neuro-Oncology* with 111. Among the top ten most productive journals, three had impact factors exceeding 10, and six were placed in the Q1 category according to Journal Citation Reports (JCR). The most frequently cited journals were *Clinical Cancer Research* with 13,201 citations, *Neuro-Oncology* with 13,069, *Cancer Research* with 12,440, the *New England Journal of Medicine* with 8079, and the *Journal of Clinical Oncology* with 7768. Among the top ten co-cited journals, three were cited more than 10,000 times, and seven had an impact factor of over 10. A density map effectively illustrated the most published and cited journals (Figs. 6 and 7).

**Table 4:** The top 10 journals and co-cited journals associated with the immunotherapy of GBM

Rank	Journal	Count	IF (2024)	JCR (2024)	Nature index	Co-cited journal	Citation	IF (2024)	JCR (2024)	Nature index
1	Frontiers in Immunology	268	5.7	Q1	NO	Clinical Cancer Research	13,201	10	Q1	NO
2	Frontiers in Oncology	167	3.5	Q2	NO	Neuro-Oncology	13,069	16.4	Q1	NO
3	Cancers	157	4.5	Q1	NO	Cancer Research	12,440	12.5	Q1	Yes
4	Journal of Neuro-Oncology	155	3.2	Q2	NO	New England Journal of Medicine	8079	96.2	Q1	Yes
5	Clinical Cancer Research	114	10	Q1	NO	Journal of Clinical Oncology	7768	42.1	Q1	Yes
6	Neuro-Oncology	111	16.4	Q1	NO	Nature	7433	50.5	Q1	Yes
7	Cancer Immunology Immunotherapy	102	4.6	Q1	NO	Journal of Neuro-Oncology	6628	3.2	Q2	NO
8	International Journal of Molecular Sciences	92	4.9	Q1	NO	Journal of Immunotherapy	6625	3.2	Q2	NO
9	Oncoimmunology	80	6.5	Q1	NO	Proceedings of the National Academy of Sciences of the United States of America	6217	9.4	Q1	NO
10	Journal for Immunotherapy of Cancer	68	10.3	Q1	NO	Nature Medicine	5716	58.7	Q1	Yes

Note: IF: Impact Factor; JCR: Journal Citation Reports.

### 3.5 Reference

**Figure 8:**
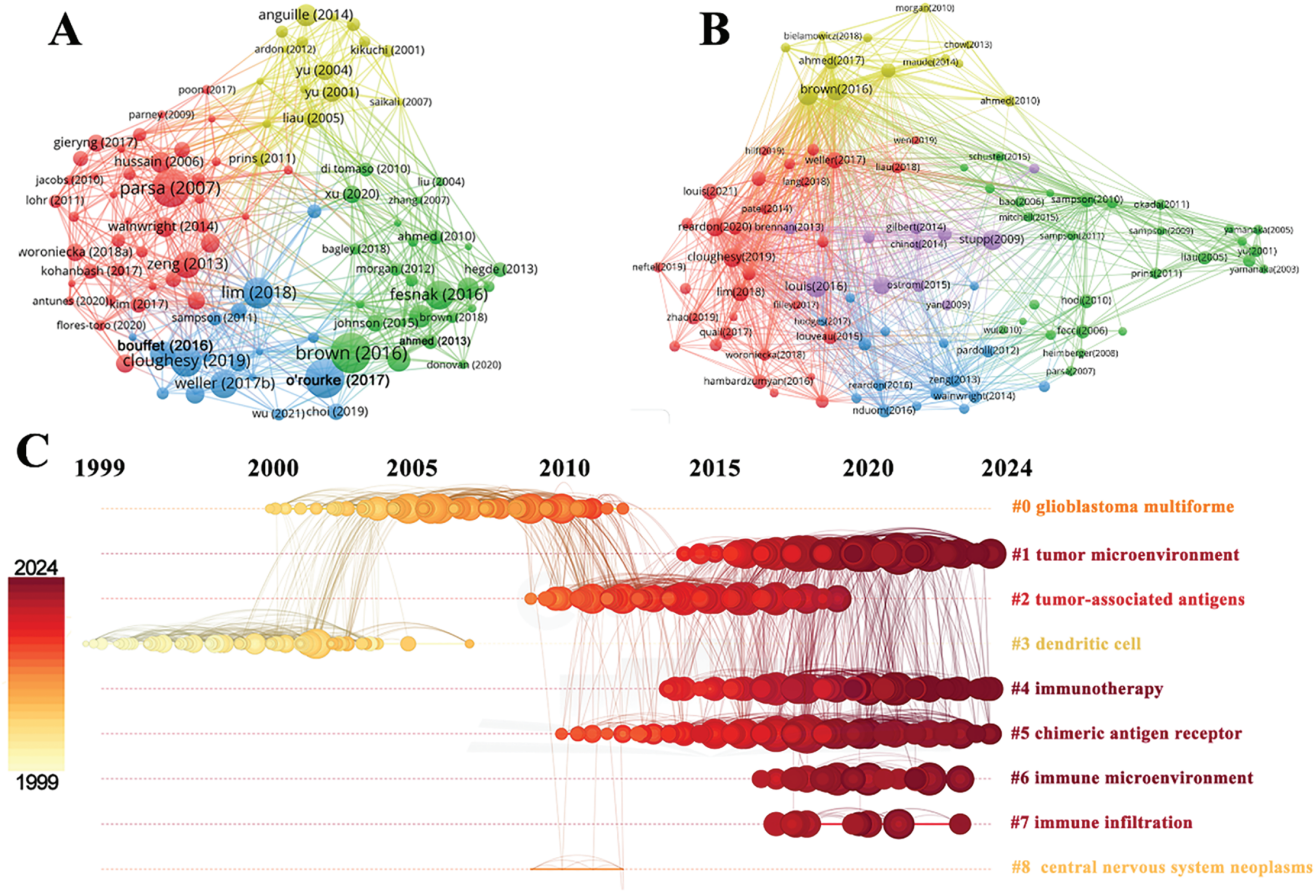
Analysis and network visualization of reference. The document’s citation strength (**A**) and co-citation strength (**B**). (**C**) Timeline distribution of the clusters. Each horizontal line represents a cluster. Node size reflects co-citation frequency, and the links between nodes indicate co-citation relationships. The node’s occurrence year is the time when they were first co-cited

**Figure 9:**
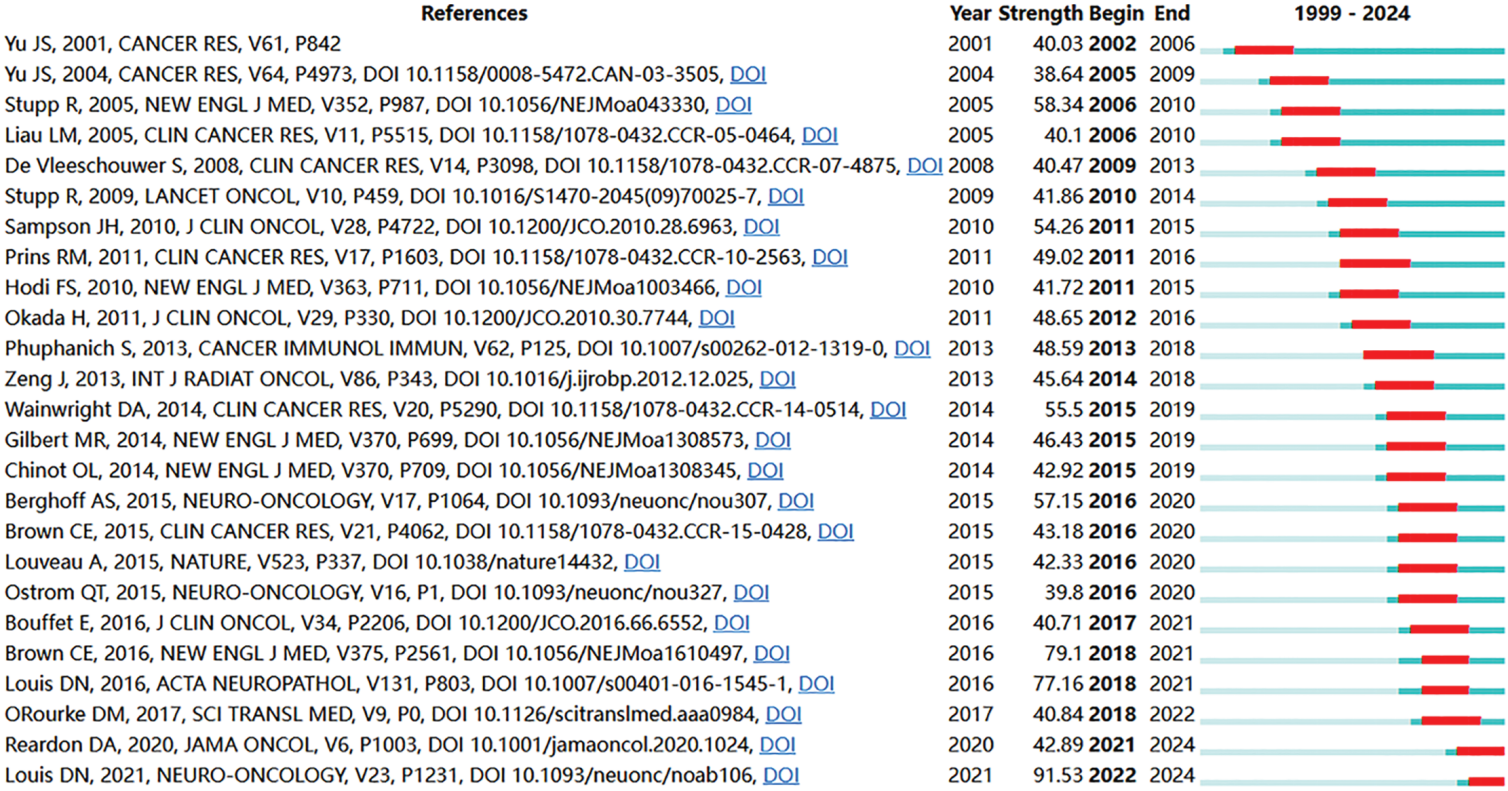
The top 25 references with the strongest citation bursts

Reference citation and co-citation analysis are widely used to assess the relevance and influence of scholarly publications. Table 5 lists the top 10 cited and co-cited references in GBM immunotherapy. In academia, the terms “cited” and “co-cited” are integral to the discourse on literature citation practices [28]. The distinction lies in their focus: “cited” refers to the frequency with which an individual document is referenced in subsequent works, highlighting its influence and relevance. In contrast, “co-cited” pertains to the collective citation patterns between documents, emphasizing the networks of intellectual connections that emerge when several documents are concurrently cited within the same body of research. In the network visualization (Fig. 8A,B), the node size represents the document’s citation and co-citation strengths. The majority of highly cited and co-cited references were published prior to 2018, indicating that current scholarly work continued to build upon a core of established knowledge. The most cited and co-cited reference was an article by Brown et al. [29] published in the *New England Journal of Medicine* in 2016 and titled “Regression of Glioblastoma after Chimeric Antigen Receptor T-Cell Therapy.” This article reported a clinical trial in which sustained 7.5-month remission was achieved in a patient with recurrent multifocal GBM. The treatment involved the innovative use of chimeric antigen receptor (CAR)-engineered T cells, which targeted the IL13 receptor alpha 2 (IL13Rα2) antigens. We further visualized the clustering timeline of the co-cited references and observed frequent mentions of tumor-associated antigens, dendritic cells (DCs), CARs, and immunotherapy (Fig. 8C). Burst analysis was also conducted on the co-cited documents, revealing significant trends and influential literature (Fig. 9). The article with the highest burst strength was by Louis et al. [30] published in *Neuro-Oncology* in 2021 and titled “The 2021 WHO Classification of Tumors of the Central Nervous System: A Summary.” This article updates the sixth version of the international standard for the classification of brain and spinal cord tumors. The article with the second-highest burst strength was also the most-cited article published by Christine E Brown in 2016. The development of more effective treatment methods has consistently been the focus of GBM research [29]. Moreover, immunotherapies based on CAR-engineered T cells have become a new trend.

**Table 5:** The top 10 cited and co-cited references in the immunotherapy of GBM

	Rank	Title	Author	Journal	Year	Citations	References
	1	Regression of glioblastoma after chimeric antigen receptor T-cell therapy	Brown CE et al.	New England Journal of Medicine	2016	1207	[29]
	2	A single dose of peripherally infused EGFRvIII-directed CAR T cells mediates antigen loss and induces adaptive resistance in patients with recurrent glioblastoma	O’Rourke DM et al.	Science Translational Medicine	2017	1148	[31]
	3	Loss of tumor suppressor PTEN function increases B7-H1expression and immunoresistance in glioma	Parsa AT et al.	Nature Medicine	2006	1097	[32]
	4	Neoadjuvant anti-PD-1 immunotherapy promotes a survival benefit with intratumoral and systemic immune responses in recurrent glioblastoma	Cloughesy TF et al.	Nature Medicine	2019	849	[33]
	5	Current state of immunotherapy for glioblastoma	Lim M et al.	Nature Reviews Clinical Oncology	2018	829	[34]
**Cited**	6	Engineered T cells: the promise and challenges of cancer immunotherapy	Fesnak AD et al.	Nature Reviews Cancer	2016	765	[35]
	7	Rindopepimut with temozolomide for patients with newly diagnosed, EGFRvIII-expressing glioblastoma (ACT IV): a randomised, double-blind, international phase 3 trial	Weller M et al.	The Lancet Oncology	2017	712	[36]
	8	Anti-PD-1 Blockade and stereotactic radiation produce long-term survival in mice with intracranial gliomas	Zeng J et al.	International Journal of Radiation Oncology, Biology, Physics	2013	677	[37]
	9	Immune checkpoint inhibition for hypermutant glioblastoma multiforme resulting from germline biallelic mismatch repair deficiency	Bouffet E et al.	Journal of Clinical Oncology	2016	619	[38]
	10	HER2-specific chimeric antigen receptor–modified virus-specific T cells for progressive glioblastoma Phase 1 dose-escalation trial	Ahmed N et al.	JAMA Oncolog	2017	555	[39]
**Co-Cited**	**Rank**	**Title**	**Author**	**Journal**	**Year**	**Co-Citations**	**References**
	1	Regression of glioblastoma after chimeric antigen receptor T-cell therapy	Brown CE et al.	New England Journal of Medicine	2016	524	[29]
	2	The 2016 world health organization classification of tumors of the central nervous system: a summary	Louis DN et al.	Acta Neuropathol	2016	506	[40]
	3	Effects of radiotherapy with concomitant and adjuvant temozolomide vs. radiotherapy alone on survival in glioblastoma in a randomised phase III study: 5-year analysis of the EORTC-NCIC trial	Stupp R et al.	The Lancet Oncology	2009	460	[41]
	4	A single dose of peripherally infused EGFRvIII-directed CAR T cells mediates antigen loss and induces adaptive resistance in patients with recurrent glioblastoma	O’Rourke DM et al.	Science Translational Medicine	2017	459	[31]
	5	Neoadjuvant anti-PD-1 immunotherapy promotes a survival benefit with intratumoral and systemic immune responses in recurrent glioblastoma	Cloughesy TF et al.	Nature Medicine	2019	425	[33]
	6	Effect of nivolumab vs. bevacizumab in patients with recurrent glioblastoma: the CheckMate 143 Phase 3 randomized clinical trial	Reardon DA et al.	JAMA Oncolog	2020	419	[42]
	7	Integrated genomic analysis identifies clinically relevant subtypes of glioblastoma characterized by abnormalities in PDGFRA, IDH1, EGFR, and NF1	Verhaak RGW et al.	Cancer Cell	2010	373	[43]
	8	Current state of immunotherapy for glioblastoma	Lim M et al.	Nature Reviews Clinical Oncology	2018	365	[34]
	9	Rindopepimut with temozolomide for patients with newly diagnosed, EGFRvIII-expressing glioblastoma (ACT IV): a randomised, double-blind, international phase 3 trial	Weller M et al.	The Lancet Oncology	2017	327	[36]
	10	The 2021 WHO classification of tumors of the central nervous system: a summary	Louis DN et al.	Neuro-oncology	2021	312	[30]

### 3.6 Key Words

**Figure 10:**
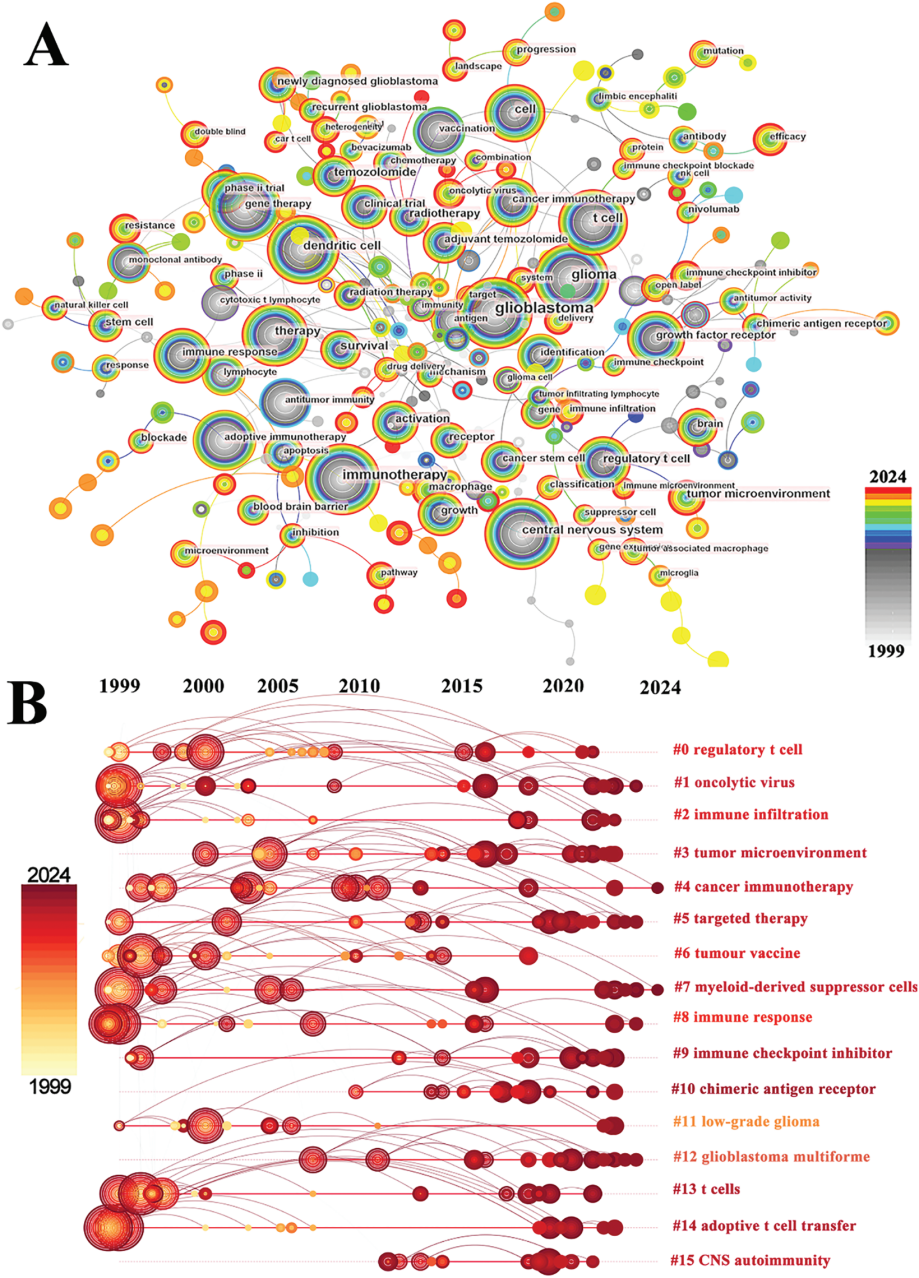
Analysis and network visualization of keywords. (**A**) The visualization map of keywords involved in immunotherapy of GBM; (**B**) Timeline distribution of cluster analysis of keywords. The node’s occurrence year is the time when they first appeared

**Figure 11:**
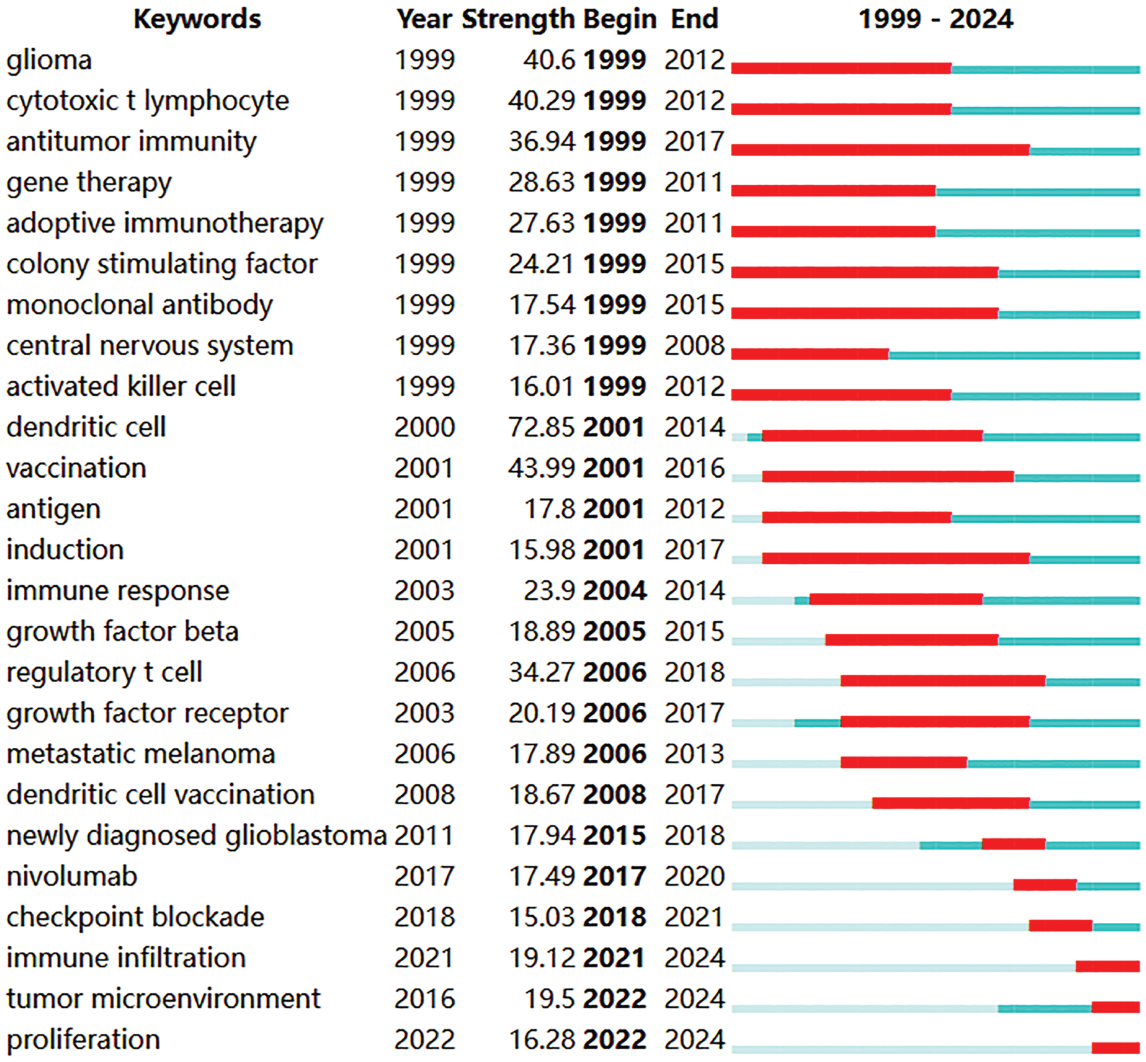
The top 25 keywords with the strongest citation bursts

Co-occurrence analysis of keywords can uncover emerging themes and developmental trends in a given field. In our study, co-occurrence analysis of keywords identified 261 nodes and 1129 links, forming 16 distinct clusters. A summary of the top ten keywords by frequency is presented in Table 6. Several core terms were identified, such as “glioblastoma” and “immunotherapy,” along with closely related concepts like “T cell,” “tumor microenvironment,” and “immune checkpoint inhibitor,” underscoring their central roles in the development of GBM immunotherapy (Fig. 10A). We performed clustering and timeline visualization analyses to trace the dynamic trajectory of research hotspots represented by keywords and explored the temporal dynamics and the rise and fall of research trends (Fig. 10B). The cluster modules identified in our analysis encompassed various topics, such as CARs, oncolytic viruses, immune infiltration, and TME. Based on the timeline visualization, researchers began analyzing the immune characteristics of GBMs and preliminarily explored the potential of immunotherapy as early as 1999. Subsequent studies further investigated the immune features of GBM-TME and developed novel immunotherapeutic approaches, including CARs, oncolytic viruses, adoptive T-cell transfer, tumor vaccines, and immune checkpoint inhibitors (ICIs). To further identify research hotspots and emerging trends in GBM immunotherapy, a keyword burst detection analysis was performed. The top 25 keywords exhibiting the most significant citation bursts are shown in Fig. 11. “Dendritic cell” exhibits the highest burst strength, rated at 72.85, and has sustained its research prominence for 15 years. We also observed that “tumor microenvironment” (since 2016), “immune infiltration” (since 2021), and “proliferation” (from 2022) have emerged as prominent recent research areas, suggesting that the TME, immune infiltration, and proliferation may become new research hotspots in this field.

**Table 6:** The top 10 keywords associated with the immunotherapy of GBM

Rank	Keyword	Count
1	Glioblastoma	2552
2	Immunotherapy	1035
3	Glioma	861
4	t cell	585
5	Central nervous system	498
6	Dendritic cell	495
7	Cell	471
8	Survival	469
9	Therapy	445
10	Tumor microenvironment	396

### 3.7 Chimeric Antigen Receptor T-Cell, Engineered T-Cell Receptor T Cell and Glioblastoma

**Figure 12:**
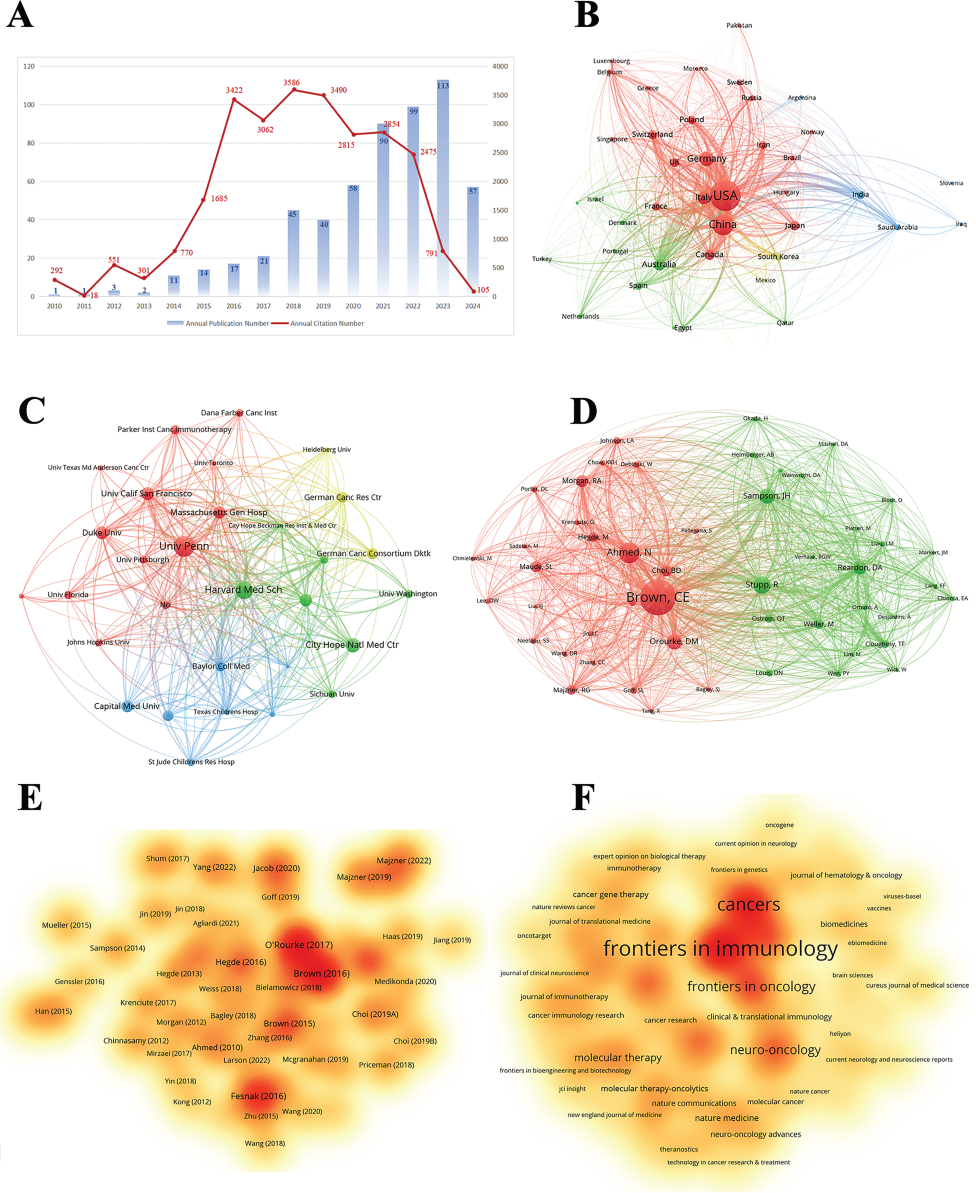
Analysis and network visualization of research on Chimeric antigen receptor T-Cell (CAR-T), engineered T-cell receptor T cell (TCR-T) and GBM. (**A**) Trends in the growth of publications; (**B**) The co-occurrence map of countries/regions; (**C**) The co-occurrence map of institutions; (**D**) The visualization map of authors; (**E**) The density map of references; (**F**) The density map of journals

Based on the aforementioned findings and emerging research focusing on CARs in GBM immunotherapy, we aimed to further explore current studies concerning Chimeric Antigen Receptor T-Cells (CAR-T), engineered T-Cell Receptor T-Cells (TCR-T), and their application in GBM treatment. Using the refined search strategy detailed in the Section 2.1, 572 studies related to CAR-T cells, TCR-T cells, and GBMs were included in subsequent analyses. Globally, this field exhibited a consistent upward trend in publication output since 2010 (Fig. 12A). The inaugural article in this domain was published in 2010 in *Clinical Cancer Research* by Ahmed and Nabil, titled “HER2-Specific T Cells Target Primary Glioblastoma Stem Cells and Induce Regression of Autologous Experimental Tumors”. Human epidermal growth factor receptor 2 (HER2)-specific CAR-T cells generated from patients with GBM effectively targeted HER2-positive GBM, suggesting a promising immunotherapeutic approach for GBM [44]. The USA led the active participation in this field (324 publications, 20,674 Citations), followed by China (120 publications, 3805 Citations) and Germany (47 publications, 2840 Citations) (Table 7, Fig. 12B). The top 10 institutions are also concentrated in the USA. The University of Pennsylvania had the highest number (40 publications, 4896 Citations), followed by Harvard Medical School (33 publications, 1900 Citations) and the City of Hope National Medical Center (26 publications, 1349 Citations) (Table 8, Fig. 12C). Christine E Brown, from the City of Hope National Medical Center, was the most published and co-cited author (25 publications, 841 Citations) (Table 9, Fig. 12D). Furthermore, the article titled “Regression of Glioblastoma after Chimeric Antigen Receptor T-Cell Therapy,” published by Christine E Brown in the *New England Journal of Medicine*, was one of the most highly cited articles (Table 10, Fig. 12E). Brown’s research program is highly translational and encompasses four ongoing clinical trials on CAR-T therapy for GBM (NCT04003649, NCT04661384, NCT04214392, and NCT03389230) [45]. The leading journal in terms of publication volume was *Frontiers in Immunology*, which published 42 articles on this topic. Additionally, the most co-cited journal was *Clinical Cancer Research* (2847 Citations) (Table 11, Fig. 12F).

**Table 7:** The top 10 countries contributing to publication in the CAR/TCR-T cell of GBM

Rank	Country	Publications	Citations	Citation Per Publication
1	USA	324	20,674	63.81
2	China	120	3805	31.71
3	Germany	47	2840	60.43
4	Italy	35	711	20.31
5	Australia	27	422	15.63
6	Canada	20	1133	56.65
7	Japan	17	397	23.35
8	Switzerland	15	746	49.73
9	UK	15	358	23.87
10	Poland	12	186	15.50

Note: CAR: Chimeric Antigen Receptor; TCR-T: engineered T-Cell Receptor T-Cells.

**Table 8:** The top 10 institutions contributing to publications in the CAR/TCR-T cell of GBM

Rank	Institution	Country	Publications	Citations	Citation per publication
1	University of Pennsylvania	USA	40	4896	122.40
2	Harvard Medical School	USA	33	1900	57.58
3	City of Hope National Medical Center	USA	26	1349	51.88
4	Duke university	USA	23	1427	62.04
5	University of California, San Francisco	USA	23	2029	88.22
6	Stanford University	USA	22	1504	68.36
7	Capital Medical University	China	21	230	10.95
8	Massachusetts General Hospital	USA	21	2452	116.76
9	German Cancer Consortium (DKTK)	German	19	1170	61.58
10	German Cancer Research Center	German	18	673	37.39

Note: CAR: Chimeric Antigen Receptor; TCR-T: engineered T-Cell Receptor T-Cells.

**Table 9:** The top 10 authors and co-cited authors in the CAR/TCR-T cell of GBM

Rank	Author	Count	Location	Co-cited author	Citation
1	Christine E Brown	25	USA	Christine E Brown	841
2	Okada Hideho	20	USA	Ahmed Nabil	444
3	Forman Stephen J	18	USA	Stupp Roger	361
4	Starr Renate	16	USA	Donald M O’Rourke	315
5	Donald M O’Rourke	15	USA	Sampson John H	304
6	Gottschalk Stephen	14	USA	Reardon David A	267
7	Sampson John H	14	USA	Morgan Richard A	233
8	Badie Behnam	13	USA	Choi Bryan D	220
9	June Carl H	13	USA	Hegde Meenakshi	187
10	Aguilar Brenda	12	USA	Weller Michael	184

Note: CAR: Chimeric Antigen Receptor; TCR-T: engineered T-Cell Receptor T-Cells.

**Table 10:** The top 10 cited references in the CAR/TCR-T cell of GBM

Rank	Title	Author	Journal	Year	Citations	References
1	Regression of glioblastoma after chimeric antigen receptor T-cell therapy	Brown CE et al.	New England Journal of Medicine	2016	1207	[29]
2	A single dose of peripherally infused EGFRvIII-directed CAR T cells mediates antigen loss and induces adaptive resistance in patients with recurrent glioblastoma	O’Rourke DM et al.	Science Translational Medicine	2017	1148	[31]
3	Engineered T cells: the promise and challenges of cancer immunotherapy	Fesnak AD et al.	Nature Reviews Cancer	2016	765	[35]
4	HER2-specific chimeric antigen receptor–modified virus-specific T cells for progressive glioblastoma: a Phase 1 dose-escalation trial	Ahmed N et al.	JAMA Oncolog	2017	555	[39]
5	Bioactivity and safety of IL13Rα2-redirected chimeric antigen receptor CD8^+^ T cells in patients with recurrent glioblastoma	Brown CE et al.	Clinical Cancer Research	2015	527	[46]
6	A patient-derived glioblastoma organoid model and biobank recapitulates inter- and intra-tumoral heterogeneity	Jacob F et al.	Cell	2020	502	[47]
7	Tandem CAR T cells targeting HER2 and IL13Rα2 mitigate tumor antigen escape	Hegde M et al.	Journal of Clinical Investigation	2016	481	[48]
8	CAR T cells targeting B7-H3, a pan-cancer antigen, demonstrate potent preclinical activity against pediatric solid tumors and brain tumors	Majzner RG et al.	Clinical Cancer Research	2019	360	[49]
9	GD2-CAR T cell therapy for H3K27M-mutated diffuse midline gliomas	Majzner RG et al.	Nature	2022	360	[50]
10	CAR-T cells secreting BiTEs circumvent antigen escape without detectable toxicity	Choi BD et al.	Nature Biotechnology	2019	338	[51]

Note: CAR: Chimeric Antigen Receptor; TCR-T: engineered T-Cell Receptor T-Cells.

**Table 11:** The top 10 journals and co-cited journals associated with the CAR/TCR-T of GBM

Rank	Journal	Count	IF (2024)	JCR (2024)	Co-cited journal	Citation	IF (2024)	JCR (2024)
1	Frontiers in Immunology	42	5.7	Q1	Clinical Cancer Research	2847	10	Q1
2	Cancers	33	4.5	Q1	Neuro-Oncology	2559	16.4	Q1
3	Frontiers In Oncology	20	3.5	Q2	New England Journal of Medicine	1905	96.2	Q1
4	International Journal of Molecular Sciences	19	4.9	Q1	Cancer Research	1777	12.5	Q1
5	Neuro-Oncology	17	16.4	Q1	Journal of Clinical Oncology	1285	42.1	Q1
6	Clinical Cancer Research	16	10	Q1	Molecular Therapy	1239	12.1	Q1
7	Journal For Immunotherapy of Cancer	15	10.3	Q1	Nature	1217	50.5	Q1
8	Molecular Therapy	11	12.1	Q1	Nature Medicine	1160	58.7	Q1
9	Science Translational Medicine	9	15.8	Q1	Blood	1092	21	Q1
10	Cells	8	5.1	Q1	Science Translational Medicine	1086	15.8	Q1

Note: CAR: Chimeric Antigen Receptor; TCR-T: engineered T-Cell Receptor T-Cells.

## 4 Discussion

Among the novel therapeutic approaches, immunotherapy continues to be a potentially effective treatment option, especially for GBMs. In this study, we analyzed literature on GBM immunotherapy published since 1999 using bibliometric techniques. We provide an in-depth examination of publication trends, geographical distribution, collaborative networks at the international and institutional levels, and key research focal points. This comprehensive analysis aimed to guide future scholarly endeavors in the field of GBM immunotherapy.

### 4.1 Trend of Publications

The number of publications on GBM immunotherapy has increased dramatically since 2018, with a peak in publication activity in 2022. This significant surge reflects growing interest in the field, which has been particularly notable since 2018. This surge in interest is primarily attributed to the 2018 Nobel Prize in Physiology or Medicine, which honored two pioneering immunologists, James P Allison from the USA and Tasuku Honjo from Japan. They are recognized for their seminal contribution to cancer therapy via negative immune regulation [52]. Consequently, the investigation of immunotherapeutic strategies for GBM has expanded into a prevalent area of research and has become a central focus of inquiry in subsequent years.

### 4.2 Countries/Institutions and Their Cooperation

The role of immunotherapy in GBM has been investigated in 96 countries. The USA, China, Germany, Italy, and Japan led this research, with the USA and China being the most prominent countries. Notably, China’s rapid development and significant influence in this field have been evident since 2021. In China, most scientific research is conducted in public universities, where principal investigators are typically professors who are reliant on government-funded grants, such as those from the National Natural Science Foundation of China. Consequently, research directions often aligned with national policy priorities to secure sustained funding. This trend may also have been influenced by the 2018 Nobel Prize in Physiology or Medicine, as the Chinese government has vigorously promoted the development of tumor immunotherapy since 2018. This initiative led to a rapid growth in related research and clinical trials, positioning China as a major contributor to the advancement of immunotherapeutic strategies for cancer. However, the academic impact of Chinese publications, as reflected by a citation-to-publication ratio of only 1.63, indicates room for improvement in research quality. Consequently, it is important to enhance regional cooperation to amplify academic impact. Among the top ten institutions ranked by publication output, six were based in the USA and three were from China. Further analysis revealed a tendency for institutions to prefer collaboration within their own countries. This insight underscores the importance of strengthening cross-institutional collaboration and breaking down academic silos to foster a more integrated global research community.

### 4.3 Citation Information

The academic influence of authors is often measured by their co-citation frequency, which reflects their recognition and impact within the scientific community. According to Tables 3 and 4, six of the top ten authors were from the USA, and one was from China. Although Chinese scholars have invested significantly in this research, there is a need to enhance the depth and quality of their studies. Collaboration with American scholars could potentially increase the quality of the research output. Similarly, a journal’s prestige is often assessed based on its co-citation count. Five of the top ten most co-cited journals are listed on the Nature Index. Notably, the *New England Journal of Medicine* was cited 8079 times and had the highest impact factor among the top ten journals, rated at 96.2.

Reference citations and co-citations are two important indicators of an article’s impact. According to Table 5, the most cited and co-cited reference is “Regression of Glioblastoma after CAR T-Cell Therapy,” published in the *New England Journal of Medicine* by Christine E Brown, posited that CAR-T cells, a form of immunotherapy, represented a promising new strategy for treating GBM [29]. CAR-T therapy has rapidly evolved as a leading adoptive immunotherapeutic approach. CAR-T therapy initially achieved substantial clinical success in hematologic malignancies and is now being actively explored for solid tumors, including GBM [53]. The extensive literature on CAR-T therapy for GBM indicates that CAR-T therapy has emerged as a primary direction for the future development of GBM immunotherapy, surpassing traditional immunotherapeutic approaches such as immune checkpoint blockade and monoclonal antibodies.

### 4.4 Research Hotspots: CAR-T against GBM

Identifying research hotspots and frontiers is essential to understanding the evolution of a field. Reference analyses revealed that CAR-T cells may be a rapidly developing topic in GBM immunotherapy. To further explore popular topics and trends in the field, we performed a keyword analysis. As depicted in Table 6, “T cell” was one of the most frequently occurring terms among the top ten keywords. Further clustering and timeline visualization analyses indicated that CAR and adoptive T cell transfer were hot topics and trends in GBM immunotherapy (Fig. 10B). We observed a notable discrepancy between our findings and those of previous studies. Zhou et al. suggested that microglia and their polarization represent emerging research directions that warrant greater consideration for GBM immunotherapy [54]. Similarly, Zhang et al. highlighted the significance of Programmed Death protein-1 (PD-1) and nivolumab in current research on glioma immunotherapy [55]. Yuan et al. argued that ICIs have emerged as a focal point and hot topic in GBM immunotherapy research [56]. Conversely, Zhang et al. posited that future research should concentrate on the TME, cancer vaccines, epidermal growth factor receptor (EGFR), and interleukin-13 receptor alpha 2 (IL-13Rα2) [57]. The differences in outcomes may be linked to variations in search terms. Compared to other studies, our research did not introduce any biased search terms, such as pembrolizumab [55], PD-1 [56] and cytokines [57] in the data collection phase. Our search strategy sufficiently encompassed all the literature related to GBM immunotherapy without any bias toward specific immunotherapeutic methods. Consequently, our data are more representative and better aligned with the current academic forefront (Tables 12 and 13).

**Table 12:** The difference between our work and others

Group	Theme	Data	Time	Search terms	Conclusion
Our work	Bibliometric analysis of immunotherapies in GBM	5038 papers	1999–2024	(“Immunotherapeutic” OR “immunotherapy”) AND TS = (“glioblastoma” OR“GBM”OR“glioblastoma”)	Our analysis suggests that CAR-T therapy is the predominant research focus in GBM immunotherapy. However, clinical translation remains limited by glioblastoma-specific barriers including immunosuppressive TME, antigen heterogeneity, and evasion mechanisms. Emerging non-T-cell approaches (CAR-NK, CAR-M) and CMV-TCR T cells show growing traction as alternative strategies to address CAR-T limitations.
PMID: 39175478		1613 papers	2012–2022	(glioblastoma* OR “glioblastoma multiform*” OR “malignant glioma” OR “brain cancer” OR gliosarcoma OR spongioblastoma) AND TS = (Immunotherapy OR Immunotherapies OR immunotherapeutic)	They suggest that immunotherapy is currently a novel treatment strategy for GBM that has attracted much attention. They prospect that it is necessary to strengthen cooperation and exchanges between countries and institutions towards relevant research to promote the development of this field in the future. While there’s much to commend, it’s worth noting that they do not suggest concrete next steps for researchers based on the bibliometric trends in their research.
PMID: 38293697		Top 100 most influential papers	2003–2022	(“glioma*” OR “astrocytoma” OR “glioblastoma” OR “ependymoma” OR “ganglioglioma” OR “gliosarcoma” OR “medulloblastoma” OR “oligodendroglioma” OR “GBM” OR “oligoastrocytoma” OR “glial cell tumor*”) AND TS = (“immunotherap*” OR “ICI” OR “ICIs” OR “CPI” OR “immune-checkpoint inhibitor*” OR “immune checkpoint inhibitor*” OR “immune-checkpoint blockade*” OR “immune checkpoint blockade*” OR “CAR-T” OR “CAR T” OR “chimeric antigen receptor T-cell*” OR “chimeric antigen receptor T cell*” OR “PD-1” OR “cytotoxic T lymphocyte-associated antigen-4” OR “CTLA-4” OR “cytokine therapy” OR “vaccine*” OR “PD-L1” OR “adoptive cell transfer therapy” OR “Adoptive cell therapy”)	They suggest that microglia and their polarization represent emerging research directions that warrant greater consideration in glioblastoma immunotherapy

**Table 13:** Previous bibliometric analysis of immunotherapies in GBM

Group	Theme	Data	Time	Search terms	Conclusion
PMID: 37867521	Bibliometric analysis of immunotherapies in GBM	4910 papers	2000–2023	TS = (ipilimumab) OR TS = (pembrolizumab) OR TS = (nivolumab) OR TS = (tremelimumab) OR TS = (immune checkpoint blockade) OR TS = (*immunotherapy) OR TS = (vaccine) OR TS = (CAR-T) OR TS = (immune checkpoint inhibitor) OR TS = (PD-1) OR TS = (PD-L1) OR TS = (CTLA-4) OR TS = (LAG-3)) AND (TS = (glioma*) OR TS = (neurolipocytoma) OR TS = (neurospongioma) OR TS = (neuroglioma*) OR TS = (glioblastoma*) OR TS = (GBM) OR TS = (gliosarcoma) OR TS = (astrocytoma)	They have underscored the significance of Programmed Death-1 (PD-1) and the drug nivolumab in current research on glioma immunotherapy
PMID: 37671057		1929 papers	1990–2023	[TI = (“glioma”) OR AK = (“glioma”) OR TI = (“malignant glioma”) OR AK = (“malignant glioma”) OR TI = (“high grade glioma”) OR AK = (“high grade glioma”) OR TI = (“glioblastoma”) OR AK = (“glioblastoma”)] AND [T2 = (“immunotherapy*”) OR AK = (“immunotherapy*”) OR T2 = (“immune NEAR/2 therap*”) OR AK = (“immune NEAR/2 therap*”) OR T2 = (checkpoint inhibitor) OR AK = (checkpoint inhibitor) OR T2 = (PD-1) OR AK = (PD-1) OR T2 = (PD-L1) OR AK = (PD-L1) OR TI = (nivolumab) OR AK = (nivolumab) OR TI = (pembrolizumab) OR AK = (pembrolizumab) OR T2 = (CAR-T) OR AK = (CAR-T) OR TI = (chimeric antigen receptor T-cell therapy) OR AK = (chimeric antigen receptor T-cell therapy) OR T2 = (dendritic cell vaccines) OR AK = (dendritic cell vaccines) OR T2 = (viral therapy) OR AK = (viral therapy)]	They have argued that immune-checkpoint inhibitors (ICIs) have emerged as a focal point and a hot topic in glioblastoma immunotherapy research
PMID: 38259287		1529 papers	2000–2023	(Cytokines) AND (Glioma) AND (Immunotherapy)	They have posited that future research is likely to concentrate on the tumor microenvironment, cancer vaccines, epidermal growth factor receptor (EGFR), and interleukin-13 receptor alpha 2 (IL-13Rα2)

T cells originate from bone marrow-derived lymphoid stem cells. Subsequently, T cells are disseminated to the immune organs and tissues throughout the body via the lymphatic and circulatory systems [58]. T cells specifically recognize “non-me” single or tumor neoantigen peptides presented through T cell receptors, and rapidly trigger the immune function of T cells to kill target cells [59,60]. CAR-T cells are T lymphocytes obtained from the blood of a patient that are modified to express CARs. These synthetic receptors redirect T cells to target surface antigens on cancer cells [61]. Upon engagement of the tumor antigen with the CAR construct, T cells are activated and proliferate. This process is accompanied by the release of cytokines and cytolytic degranulation of perforin and granzyme, thereby enabling targeted elimination of tumor cells [62,63]. CAR-T is now used to treat GBM and its targets mainly include HER2, IL-13Rα2, EGFRvIII, CD70, EphA2, B7-H3, and chlorotoxin [29,64,65]. HER2, a cell membrane receptor with tyrosine kinase activity, is overexpressed in many types of cancers, including GBM [66]. In contrast, HER2 expression is absent in normal neuronal and glial tissues [39]. In 2010, HER2-specific CAR-T cells were developed for GBM treatment and demonstrated potent antitumor activity [29]. The IL-13Rα2 is overexpressed in 75% of GBM malignant cells and is involved in the activation of the phosphatidylinositol 3-kinase/AKT/mammalian target of rapamycin pathway [67]. It has been reported that a patient with recurrent GBM was successfully treated with CAR-T targeting IL-13Rα2 [29]. Regrettably, there was subsequent disease progression, attributed to a phenomenon known as antigen escape, which resulted in a gradual reduction in the expression levels of IL-13Rα2 on the surface of GBM cells. Several constraints undermine the potency of CAR-T therapy for GBM. For example, the temporal and spatial heterogeneity of the target antigen expression in GBM cells poses a significant impediment to the efficacy of CAR-T therapy. CAR-T cells combined with an immune checkpoint blockade may be key to overcoming this drawback. There have been clinical trials attempting to verify whether IL-13Rα2-targeted CAR-T therapy combined with nivolumab and ipilimumab is more effective in treating patients with GBM (NCT04003649) [68].

### 4.5 The Current State and Challenges of Other Immunotherapy Strategies for Glioblastoma

#### 4.5.1 Immune Checkpoint Blockade Therapy

Immune checkpoints represent a category of regulatory proteins expressed on the surface of specific immune cells, predominantly T cells, which play pivotal roles in preserving immune system equilibrium and averting autoimmune reactions [69,70]. However, these immune checkpoints can be hijacked by malignancies to escape surveillance and inhibit host defenses [71,72]. Various immune checkpoints have been identified over the past few decades. These include PD-1 and programmed death ligand 1 (PD-L1), cytotoxic T lymphocyte-associated protein 4 (CTLA-4), lymphocyte activation gene 3 (LAG-3), T cell immunoreceptor with immunoglobulin and immunoreceptor tyrosine-based inhibitory motif domains (TIGIT), T cell immunoglobulin and mucin domain 3 (TIM-3), V-domain Ig suppressor of T cell activation, and indoleamine 2,3-dioxygenase 1 (IDO1) [10]. Monoclonal antibodies targeting these immune checkpoints, collectively termed ICIs, have demonstrated substantial efficacy in the treatment of several advanced cancers, including melanoma, non-small cell lung cancer, colorectal cancer, gastric cancer, and hepatocellular carcinoma [73]. However, numerous clinical trials have indicated that ICIs demonstrate limited efficacy against GBM [74]. However, numerous clinical trials have indicated that ICIs have limited clinical benefits in patients with GBM [74]. For example, nivolumab (an anti-PD-1 antibody) did not improve the median overall survival of patients with GBM (NCT02617589 and NCT02667587) [75,76]. Pembrolizumab (another type of anti-PD-1 antibody) also exhibited restricted clinical efficacy in patients with recurrent GBM, whether administered as monotherapy or in conjunction with bevacizumab [77–79]. In addition, ipilimumab (an anti-CTLA-4 antibody) did not improve the prognosis of patients with GBM in a phase II clinical trial [80]. Moreover, the combination of ipilimumab and nivolumab did not bring clinical benefits to patients with GBM, but instead increased immune-related toxicity [81]. Several emerging targets, including LAG-3, TIM-3, TIGIT, and IDO1, are being actively explored for the treatment of GBM. To date, no clinical trials have demonstrated that these emerging targeted inhibitors can effectively treat GBM. The limited efficacy of ICIs may be partly attributed to the fact that large molecules generally cannot cross the BBB [82]. In addition, the clinical response of GBM to ICIs is associated with specific molecular alterations, immune expression signatures, and immune infiltration, which reflect the clonal evolution of the tumor during treatment [83]. This indicates that, although GBM may initially be sensitive to ICIs, its ability to adapt can ultimately lead to the development of resistance. Furthermore, tumor-associated macrophages are the main immune cells in the GBM immune microenvironment [84,85]. Thus, ICIs targeting T cells have demonstrated limited efficacy in GBM treatment.

#### 4.5.2 Oncolytic Virotherapy

Oncolytic viruses are replication-competent viral agents capable of selectively propagating within and lysing tumor cells. They induce lysis of target cells, resulting in the release of various immunostimulatory signals into the TME, including viral pathogen-associated molecular patterns, damage-associated molecular patterns, tumor-associated antigens, and proinflammatory cytokines. This cascade of events triggers antitumor immune responses of cytotoxic CD8^+^ T cells, resulting in transformation of the TME from an immunologically “cold” to a “hot” environment [86]. Herpes simplex virus (HSV), a neurotropic virus belonging to the Herpesviridae family, was the first oncolytic virus used to treat GBM [87]. HSV-1716 is a genetically engineered HSV designed to selectively replicate in actively dividing tumor cells through the deletion of the *RL1* gene encoding ICP34.5 proteins. Multiple phase I clinical trials have demonstrated the safety of HSV-1716 for the treatment of GBM [88–90]. However, there is insufficient evidence demonstrating its effectiveness in the treatment of GBM. HSV-G207, a second-generation oncolytic HSV, contains deletions in both *RL1* copies and an inactivated insertion in UL39. Several clinical trials have demonstrated the safety of HSV-G207 in patients with GBM [91–93]. There is evidence that patients with recurrent or progressive pediatric high-grade gliomas are sensitive to HSV-G207 [94]. HSV-G47Δ, a third-generation oncolytic herpes simplex virus further engineered from HSV-G207, carries an additional α47 gene deletion. This modification enhances viral replication and promotes tumor-specific immunity through increased major histocompatibility complex (MHC) class I molecule expression [95]. A phase II trial reported that intratumoral injection of HSV-G47Δ markedly increased the number of tumor-infiltrating lymphocytes and prolonged the survival of patients with GBM [96]. PVSRIPO is a non-pathogenic virus engineered by combining poliovirus and rhinovirus. It selectively targets neoplastic cells via the poliovirus receptor CD155, which is abundantly expressed on GBM cells. In several phase I clinical trials, intratumoral infusion of PVSRIPO in patients with recurrent GBM did not induce neurotoxicity. Moreover, these patients exhibited higher survival rates at both 24 and 36 months than those of historical controls [97,98]. DNX-2401 is a genetically modified oncolytic adenovirus. It features a 24-base pair deletion in the *E1A* gene, which allows selective replication in cancer cells with Rb pathway defects, while reducing replication in normal cells. Additionally, the incorporation of an Arg-Gly-Asp (RGD) motif improves its binding to GBM cells through *αV* integrins [99]. DNX-2401 was delivered via stereotactic injection to patients with GBM and demonstrated a favorable safety profile [100,101]. Moreover, a phase I/II clinical trial assessing the combination of DNX-2401 and the anti-PD-1 antibody pembrolizumab in patients with recurrent GBM revealed that this combination therapy was safe and conferred a significant survival benefit to certain patients [99]. Notably, the development of most oncolytic viral therapeutic products for GBM is still in the clinical trial stage. Thus far, HSV-G47Δ is the sole therapeutic agent to receive conditional approval from Japan’s Pharmaceuticals and Medical Devices Agency for brain tumor therapy. Oncolytic viruses are predominantly administered via intratumoral injection, which is feasible only for superficial or readily accessible tumors. However, this approach does not address the therapeutic needs of intracranial tumors. Although intravenous injections can systemically disseminate oncolytic viruses, rapid clearance of the virus from the bloodstream often results in suboptimal concentrations within tumor tissues. Secondly, most oncolytic viruses face challenges in penetrating the BBB, which restricts their utility in the treatment of GBM. Although the ability of oncolytic adenoviruses to cross the BBB can be enhanced by coating them with a cell membrane, this approach is still in the experimental phase [102].

#### 4.5.3 Vaccine Therapy

Cancer vaccines are designed to harness the adaptive immune system of patients against specific endogenous and exogenous tumor antigens, thereby enabling either prophylactic or therapeutic outcomes [103]. Cancer vaccines are primarily categorized based on two antigen selection strategies: “predefined” and “anonymous” antigens. Predefined antigens comprise public tumor antigens commonly expressed in many tumors, as well as patient-specific neoantigens derived from individual genetic alterations. In contrast, anonymous antigens represent molecular targets that remain unidentified and can be either externally loaded onto antigen-presenting cells (APCs) under laboratory conditions or designed to stimulate resident APCs directly within the tissue following administration [104]. Upon vaccination, antigenic epitopes are processed and displayed on APC surfaces to prime naïve T cells. Activated T cells then traffic to tumor sites, where they mediate cytotoxic effects, leading to tumor shrinkage, and concurrently generate durable immunosurveillance mechanisms that help prevent recurrence [105]. Currently, four major vaccine platforms are being evaluated for GBM therapy: peptide-based, nucleic acid (DNA/RNA), DC, and viral vector-based vaccines [106].

Peptide vaccines employ synthetic peptides to mimic the epitopes of tumor-specific or tumor-associated antigens, thereby eliciting novel or augmented tumor-specific T cell responses [107]. Survivin, an anti-apoptotic protein, is significantly upregulated in GBM and remains largely undetectable in normal brain parenchyma [108]. The safety of the SurVaxM vaccine targeting survivin was evaluated in a phase I clinical trial involving nine patients with survivin-positive recurrent GBM [109]. Moreover, phase IIa clinical trials have demonstrated that SurVaxM plus adjuvant temozolomide provides clinical benefits to patients with newly diagnosed GBM [110]. IMA950 is a multiple-peptide vaccine designed for GBM treatment comprising 11 tumor-associated peptides displayed on human leukocyte antigen (HLA) surface receptors in most GBM tumors [111]. A phase I study evaluated the safety and immunogenicity of IMA950 in combination with temozolomide, which generated additional clinical benefits in at least 30% of patients with newly diagnosed GBM [112]. Similarly, it has been demonstrated that IMA950 combined with immunostimulant poly-ICLC improves the prognosis of patients with newly diagnosed GBM [113]. However, the IMA950 peptide vaccine showed no clinical benefits in patients with recurrent GBM [114]. CDX-110 is a peptide-based vaccine containing a 13–amino acid epitope, specifically designed to elicit an immune response against the EGFRvIII antigen variant [115]. However, the CDX-110 peptide vaccine did not increase the survival of patients with GBM in a phase III clinical trial [36].

DC vaccines use patient-derived DCs to prime antitumor immune responses by presenting tumor-associated antigens to T cells [116]. DC vaccines are typically prepared using patient-specific DCs, which are obtained either by direct isolation from peripheral blood or through cytokine-induced differentiation of monocytes or CD34^+^ hematopoietic stem cells. DCs loaded with tumor antigens stimulate CD4^+^ T cell responses via peptide–MHC class II presentation and activate CD8^+^ T cells through the cross-presentation of MHC class I molecules [117]. DCVax-L, an autologous DC vaccine activated by an autologous tumor lysate, has been shown to be beneficial and safe for patients with GBM, potentially extending survival [118,119]. ICT-107 is a DC vaccine derived from autologous or monocyte-derived DCs, which target six antigens on tumor cells, including glycoprotein 100, IL-13Rα2, tyrosinase-related protein-2, antigen isolated from immunoselected melano-ma-2, the HLA-A2-restricted human EGFR-2 (HER2/neu), and the HLA-A1-restricted melanoma-associated antigen-1 [120]. Although ICT-107 exhibits favorable safety and immunogenicity in patients newly diagnosed with GBM, it does not significantly improve the median survival [120]. The cytomegalovirus (CMV)-DC vaccine, also known as CMV-pp65 RNA-pulsed DC vaccine, is an autologous DC vaccine that targets the CMV matrix protein pp65. Human CMV proteins are expressed in more than 90% of GBMs [121]. Moreover, the absence of CMV protein expression in surrounding normal brain tissue offers a unique opportunity to exploit CMV antigens as tumor-specific targets. Three separate clinical trials have shown that nearly one-third of the patients with GBM receiving CMV–DC vaccines are long-term survivors [122–124].

Nucleic acid vaccines involve the delivery of plasmid vectors containing gene sequences encoding protein antigens into the host body via intramuscular injection or gene gun bombardment. These vectors enable host cells to express antigenic proteins, induce immune responses against antigenic proteins within the host cells, and prevent and treat diseases [125]. Nucleic acid-based vaccines contain both DNA and RNA. RNA vaccines deliver mRNA into host cells, particularly APCs, to produce translated peptides. RNA vaccines offer significant safety benefits owing to their rapid degradation and minimal risk of infection or insertional mutations [126]. However, no clinical trials have been conducted using RNA vaccines to treat GBM. DNA vaccines are created by inserting genes encoding tumor-associated antigens into bacterial plasmids. DNA vaccines can elicit strong CD4^+^ and CD8^+^ T cell responses by presenting antigens on MHC class I and II molecules and generating humoral immune responses [127]. VXM01 is a DNA plasmid vaccine that incorporates an attenuated strain of Salmonella typhimurium encoding murine vascular endothelial growth factor receptor-2, a protein involved in tumor angiogenesis [128,129]. In a phase I clinical trial (NCT02718443), VXM01 was well-tolerated and successfully triggered a vascular endothelial growth factor receptor-2-specific T cell immune response in patients with recurrent GBM. Importantly, patients with prolonged survival had low intratumoral PD-L1 expression, implying that the combination of VXM01 and anti-PD-L1 therapy may be advantageous [130].

Viral vector vaccines are a type of vaccine that employs viruses as vectors to deliver the genetic material of target antigens into host cells. These antigens are then expressed by the host cell machinery to synthesize antigenic proteins, which in turn stimulate immune responses [131]. In a phase IIa clinical trial, VBI-1901, a viral vector vaccine targeting two CMV antigens, glycoprotein B, and phosphoprotein 65, demonstrated potential survival benefits in patients with GBM [132].

Low tumor mutation burden (TMB) is the main challenge for GBM vaccines. CNS tumors, particularly GBM, may originate primarily from epigenetic lesions characterized by a low TMB [133,134]. However, the presentation of neoantigens is a probabilistic process contingent upon their abundance. In GBM cells, only a limited number of mutations manifest as neoepitopes capable of efficient recognition by autologous T cells [135]. Although some neoantigens may exhibit immunogenicity and are presented by APCs, a small fraction of mutations is processed into MHC-presenting neoepitopes that can be targeted by T cells. Most originate from passenger mutations and possess limited potency because tumor subclones lacking these highly antigenic tumor-specific mutant peptides evade T cell–dependent immunoselection [136,137]. Furthermore, antigen spreading, a vital mechanism underlying vaccine immunotherapy responses, is triggered by increased tumor antigen exposure following therapeutic tumor vaccination, primarily due to initial vaccine-mediated tumor lysis [138]. Thus, a smaller antigen pool resulting from a low TMB indicates that fewer immuno-genic neoantigens are exposed post-vaccination, resulting in the inhibition of antigen spreading and potentially diminishing the clinical benefits of vaccine therapy.

### 4.6 Emerging Immunotherapy Strategies for GBM

#### 4.6.1 Combined Immune Therapies Strategy against GBM

CAR-T therapy has gained considerable attention for GBM treatment, substantially broadening the scope of immunotherapy options. However, treatment-related challenges, such as antigen escape, immunoediting, and the development of adaptive resistance are frequently encountered [139]. This is often compounded by enhanced immunosuppressive mechanisms, including upregulation of PD-1 [140]. Consequently, combining CAR-T cells with other agents, such as ICIs, represents a promising avenue to counteract monotherapy-induced immunoresistance. Clinical research on CAR-T cell and ICI co-administration in GBM remains at an early stage. A recent phase I trial reported that combining EGFRvIII-CAR-T cells with the anti-PD-1 agent pembrolizumab did not improve outcomes in patients newly diagnosed with EGFRvIII-positive GBM [141]. Meanwhile, an ongoing phase I study is evaluating the safety and efficacy of IL-13Rα2–CAR-T therapy together with nivolumab (anti-PD-1) and ipilimumab (anti-CTLA-4), underscoring the interest in this multi-modal strategy for GBM [142,143].

#### 4.6.2 Next-Generation Cellular Therapeutics against GBM

Natural killer (NK) cell-based therapies have attracted growing interest in the field of cancer immunotherapy, as reflected by an increasing number of publications in recent years [144,145]. CAR-NK therapy, which involves engineering NK cells to express CARs, is a promising alternative for CAR-T cells. A key bibliometric insight is that research on CAR-NK cells emphasizes their favorable safety profile, including reduced risks of graft-vs.-host disease and cytokine release syndrome and their ability to target tumors independent of MHC restriction [146–148]. These advantages have made CAR-NK cell therapy an attractive option for treating GBM, with emerging pre-clinical studies targeting antigens such as EGFRvIII, EGFR, and HER2 [149–151]. The increasing number of publications in this field suggests that CAR-NK cells may play an increasingly important role in future GBM treatment strategies.

Similarly, the application of CAR macrophages (CAR-M) to GBM is an emerging topic as demonstrated by our bibliometric analysis. Although empirical data specific to GBM are still limited, current literature highlights the potential of CAR-M to remodel the immunosuppressive TME through phagocytosis, cytokine secretion, and antigen presentation [63,152,153]. While there is currently a lack of empirical data and no studies have been conducted on the application of CAR-M therapy for GBM, these properties endow macrophages with the potential to overcome the challenges posed by the immunosuppressive GBM TME and the limitations of CAR-T therapies.

Another evolving research direction involves TCR-T therapy, which leverages T cells engineered with exogenous T cell receptors to recognize a broader range of antigens, including viral oncoproteins. Bibliometric analysis reveals a niche but a growing body of work focused on targeting cytomegalovirus (CMV) antigens in GBM, which are present in the majority of patients [121]. A notable study by Long et al. demonstrated significant antitumor effects of CMV-specific TCR-T cells in preclinical models [154], underscoring the potential of this approach as a complementary strategy to CAR-based therapies.

## 5 Limitations

In this study, we conducted an extensive bibliometric analysis of academic reports on immunotherapy for GBM published between 1999 and 2024 using the WoSCC database and identified 5038 documents. While the Section 3 clearly presents the data, we acknowledge a lack of depth in interpreting certain trends, such as a decline in publication numbers. Therefore, we will further explore the potential reasons for fluctuations in publication trends, including changes in funding, shifts in research priorities, and possible biases related to database selection in the future. Notably, reliance solely on the WoSCC database may have introduced a selection bias, as the database predominantly includes English-language journals and may not encompass all relevant publications from regional or non-English sources. This may have affected the generalizability of the identified bibliometric and thematic distributions. Moreover, although every effort was made to exclude irrelevant and duplicate entries using a combination of automated tools and manual curation, some publications may have been misclassified or omitted. The focus on original articles and reviews also meant that emerging ideas presented in conference proceedings or preprints were not captured, potentially overlooking recent developments. Additionally, although our study excels in quantifying research activity, it does not evaluate the methodological rigor of individual studies. Consequently, these results should be interpreted in conjunction with their clinical relevance and implications for future research. Despite its limitations, this study lays a solid foundation for ongoing research in the field and highlights key issues that future studies must address.

## 6 Conclusion

This study presents an exhaustive bibliometric analysis of the literature on GBM immuno-therapy published between 1999 and 2024. Our objective was to delineate the research land-scape and identify key areas of interest using a structured bibliometric approach supplemented with visual data representation. The results indicated a sustained and growing trend in scholarly engagement in this domain. Prior to 2020, the USA consistently held the leading position in GBM immunotherapy research. However, beginning in 2021, there was a marked increase in contributions from Chinese researchers, with their publication output temporarily surpassing that of the USA. Despite this increase, the overall scholarly impact of Chinese researchers remains lower than that of their American counterparts.

Our analysis revealed that adoptive T cell therapies, particularly CAR-T therapy, not only represent the current research focus, but also delineate the principal trajectory for future advancement. The practical implications of these findings suggest that researchers should prioritize addressing the challenges associated with CAR-T cell therapy for GBM, including the immunosuppressive TME, tumor antigen heterogeneity, and mechanisms of immune evasion. Future studies should aim to enhance the efficacy of CAR-T cells by overcoming these obstacles using non-T cell-engineered immune cells and combined immunotherapeutic strategies. Additionally, the data underscore the importance of strengthened international collaboration for advancing the field. This is exemplified by the evolving patterns in research output and the critical need for global knowledge sharing to improve outcomes in patients with GBM.

In conclusion, our study provides not only an in-depth understanding of the current state of GBM immunotherapy but also actionable insights and guidance for future research. Despite these limitations, our findings establish a robust foundation for further investigations and emphasize the importance of improving the efficacy of CAR-T cell therapy, developing non-T cell-engineered immune platforms and combination treatment strategies, identifying predictive biomarkers, and fostering cross-border scientific collaboration.

## Data Availability

Data are available from the corresponding authors upon reasonable request and with the permission of the corresponding authors.

## Abbreviations

APCs: Antigen-presenting cells
BBB: Blood brain barrier
BC: Betweenness centrality
CNS: Central nervous system
CAR: Chimeric antigen receptor
CAR-T: Chimeric Antigen Receptor T-Cells
CTLA-4: Cytotoxic T-lymphocyte associated protein 4
CMV: Cytomegalovirus
DC: Dendritic cell
EGFR: Epidermal growth factor receptor
GBM: Glioblastoma
HSV: Herpes simplex virus
HLA: Human leukocyte antigen
IDO1: Indoleamine 2,3-dioxygenase 1
IL-13Rα2: Interleukin-13 receptor alpha 2
ICIs: Immune-checkpoint inhibitors
JCR: Journal Citation Reports
LAG-3: Lymphocyte activation gene 3
MGMT: O6-methylguanine-DNA methyltransferase
NK: Natural killer
PD-1: Programmed Death-1
TME: Tumor microenvironment
TTFields: Tumor Treating Fields
TCR-T: Engineered T-Cell Receptor T-Cells
TCR: T cell receptor
TIGIT: T-cell immunoreceptor with immunoglobulin and immunoreceptor tyrosine-based inhibitory motif domains
TIM-3: T-cell immunoglobulin and mucin domain 3
TMZ: Temozolomide
TAAs: Tumor-associated antigens
TMB: Tumor mutation burden
TAMs: Tumor-associated macrophages
VEGFR-2: Vascular endothelial growth factor receptor-2
WoSCC: Web of Science Core Collection
